# Potential Biomarkers for Fat from Dairy and Fish and Their Association with Cardiovascular Risk Factors: Cross-sectional Data from the LifeLines Biobank and Cohort Study

**DOI:** 10.3390/nu11051099

**Published:** 2019-05-17

**Authors:** Ilse G. Pranger, Frits A. J. Muskiet, Ido P. Kema, Cécile Singh-Povel, Stephan J. L. Bakker

**Affiliations:** 1 Department of Internal Medicine, University Medical Center Groningen and University of Groningen, Hanzeplein 1, 9700 RB Groningen, The Netherlands; s.j.l.bakker@umcg.nl; 2 Department of Laboratory Medicine, University Medical Center Groningen and University of Groningen, Hanzeplein 1, 9700 RB Groningen, The Netherlands; f.a.j.muskiet@umcg.nl (F.A.J.M.); i.p.kema@umcg.nl (I.P.K.); 3 FrieslandCampina Amersfoort, Stationsplein 4, 3818 LE Amersfoort, The Netherlands; Cecile.Singh-Povel@frieslandcampina.com

**Keywords:** dairy, fish, fatty acids, biomarkers, cardiovascular diseases, cardiovascular risk Factors, cross-sectional

## Abstract

Dairy fat intake, reflected by the biomarkers C14:0, C15:0, C17:0, trans-C16:1 (*n*-7), trans-C18:1 (*n*-7) and CLA, may have beneficial effects on cardiovascular health. It has, however, been questioned whether this association is genuine, since C15:0 and C17:0 are also biomarkers from fish. We investigated whether the above biomarkers are reliable markers for dairy fat intake in 864 healthy subjects. Subsequently, we explored the association between these biomarkers and cardiovascular risk factors. Intakes of dairy and fish were determined by Food Frequency Questionnaires FFQs. Fatty acids were analyzed in plasma triglycerides (TG) and phospholipids (PL). Median intakes of dairy and fish fat were 12.3 (8.4–17.4) g/day and 1.14 (0.53–1.75) g/day. All fatty acids, except TG C17:0, were associated with dairy fat (std.β range TG: 0.12 for C14:0 till 0.25 for C15:0 and Trans-C18:1 (*n*-7); and std.β range PL: 0.12 for C17:0 and Trans-C16:1 (*n*-7) till 0.24 for Trans-C18:1 (*n*-7) and CLA; *p* < 0.001). TG C17:0 was associated with fish fat (std.*β* = 0.08; *p* = 0.03), whereas PL C17:0 was not. Associations remained after adjustment for fish/dairy fat intake. Strongest inverse associations with biological variables were found with PL C17:0 and Trans-C18:1 (*n*-7) (Std.βs: waist circumference: −0.18, *p* < 0.001 and −0.10, *p* < 0.05; BMI: −0.17, *p* < 0.001, −0.11, *p* < 0.01; glucose: −0.10, *p* <0.01 and −0.08, *p* <0.05; high sensitive C-reactive protein (hs-CRP): −0.22, *p* < 0.001 and −0.16, *p* < 0.01; uric acid: −0.27, *p* < 0.001 and −0.24, *p* < 0.001). In conclusion, fatty acid biomarkers, except plasma TG C17:0, were associated with dairy fat intake, independent of fish fat intake. PL C17:0 and trans-C18:1 (*n*-7) were inversely associated with adiposity, diabetes, inflammation and uric acid.

## 1. Introduction

Cardiovascular diseases have become the number one cause of death worldwide [1]. Dietary and nutritional components are relevant players in the development and progression of cardiovascular diseases. Fatty acids are one of the nutritional components that may have influence on cardiovascular diseases. Whether this impact is positive or negative can for instance depend on the dietary source of the fatty acid. Earlier research has already shown that this was the case for the associations between saturated fatty acids and incident cardiovascular diseases [2,3]. For example, saturated fatty acids from meat were found to increase cardiovascular risk, while saturated fatty acids from dairy were inversely associated with cardiovascular risk [3,4,5,6,7]. Additionally, researchers have reported that trans fatty acids from processed foods, margarines, frying fast foods and commercial baked goods may increase risk for cardiovascular disease [8,9,10,11]. In contrast, trans fatty acids from dairy may actually be inversely related to cardiovascular risk factors [5]. Saturated and trans fatty acids from dairy products therefore seem to have a positive effect on cardiovascular health.

The saturated fatty acids pentadecanoic acid (C15:0), heptadecanoic acid (C17:0), and to a lesser extent myristic acid (C14:0), and the trans fatty acids trans-palmitoleic acid (trans-C16:1(*n*-7), vaccenic acid (trans-C18:1(*n*-7) and the major conjugated linoleic acid (CLA) (cis-9, trans-11 CLA) were identified as dairy fat biomarkers. Additionally, dairy fat intake, measured with the dairy fat biomarkers, was found to be inversely associated with cardiovascular risk factors [4,5,6,12,13,14,15,16]. Despite the evidence, researchers raised concerns on the association between dairy fat intake, measured by dairy fat biomarkers, and cardiovascular health, since dairy fat biomarkers may not be explicit enough for the prediction of dairy fat intake [17]. For instance, C15:0 and C17:0 were also identified as possible biomarkers for fish fat intake [18,19]. Both fish intake and fish fat intake have already been found to be inversely related to the development of cardiovascular diseases [20]. In a meta-analysis of 19 observational studies, it was found that fish consumption was associated with a risk reduction of 17% for fatal coronary heart disease [21]. Additionally, a meta-analysis with 14 randomized controlled trials demonstrated that an intervention group who consumed omega-3 fatty acid supplements (eicosapentaenoic acid (EPA) + docosahexaenoic acid (DHA), two fatty acids that are well-known biomarkers of fish intake) had an 8% reduction for cardiac death compared to the control group [22]. Because of the potential association of C15:0 and C17:0 with fish intake, researchers are concerned that the current circulating fatty acids that have been identified as dairy fat biomarkers, are not explicit enough for the prediction of dairy fat intake [19]. Therefore, inverse associations of the fatty acids with cardiovascular health may potentially not be ascribed to dairy fat intake, but fish fat intake instead.

One of the main purposes of this paper was therefore to find out whether the dairy fat biomarkers are solid biomarkers for dairy fat intake in the general population. We therefore investigated the association of the saturated fatty acids C15:0 and C17:0 with dairy fat intake, and additionally for fish fat intake. We repeated these measurements for the other dairy fat biomarkers (C14:0, trans-C16:1 (*n*-7), trans-C18:1 (*n*-7) and CLA). Secondly, we wanted to explore whether these circulating fatty acids were associated with cardiovascular risk factors, and so whether the association with cardiovascular risk factors could be ascribed to dairy fat intake, fish fat intake or both. To answer these questions, a subset from the Lifelines Biobank and Cohort study was used, a large observational cross-sectional study with initially healthy participants living in the northern part of the Netherlands.

## 2. Materials and Methods

### 2.1. Study Design and Population

The LifeLines Cohort Study is a large observational population-based cohort study and Biobank which examines the health and health-related behaviors of more than 167,000 persons [23]. The participants were recruited from the three Northern provinces of the Netherlands between 2006 and 2013. A more detailed description of the Lifelines Cohort study can be found elsewhere [24,25]. In short, the first group of participants were recruited via local general practices. Participants could indicate whether family members were interested as well. In addition, individuals who were interested in the study had the possibility to register via an online self-registration. Individuals with insufficient knowledge of the Dutch language, with severe psychiatric or physical illness, and those with limited life expectancy (<5 years) were excluded from the study. Participants (>18 years old) completed several questionnaires, including topics such as occurrence of diseases, general health, medication use, diet, physical activity and personality. Participants were invited to the Lifelines Research sites for a comprehensive health assessment. A randomly selected amount of 864 participants from the baseline cross-sectional database were included in the current study. All participants provided written consent. The Lifelines Cohort Study was conducted according to the principles of the Declaration of Helsinki and approved by the Medical ethical committee of the University Medical Center Groningen, The Netherlands.

### 2.2. Dietary Intake

To asses dietary intake in the Lifelines Cohort, a 110-item semi-quantitative baseline food frequency questionnaire (FFQ) assessing food items over the previous month was developed and validated by Wageningen University using the Dutch FFQTOOL^TM^, in which food items were selected based on the Dutch national Food Consumption Survey of 1997/1998 [26]. Seven answers categories were used to assess consumption frequency, ranging from ‘not this month’ to ‘6–7 days a week’. Portion size was estimated by fixed portion sizes (e.g., slices of bread, pieces of fruit) and commonly used household measures (e.g., cups, spoons). Energy and macronutrient intake was estimated from the FFQ data by using the Dutch food composition database of 2011 (NEVO). This study specifically looked at total dairy fat intake and total fish fat intake. Total dairy fat intake consisted of fat from cheese, milk, buttermilk, yoghurt, sweetened yoghurt drinks, custard, curd cheese, ice cream, whipped cream and porridge. Total fish fat intake consisted of fat from lean and fatty fish, including types such as salmon, herring and codfish.

### 2.3. Data on Education, Smoking Habits and General Health

Information about education, smoking and general health was collected from the self-administered questionnaire. Educational level was categorized in four groups (1. Never been to school or elementary school only, 2. Lower vocational or secondary schooling, 3. Intermediate vocational schooling or intermediate/higher secondary schooling or 4. Higher vocational schooling or university). Additionally, subjects were classified according to their smoking habits (non-smokers, former smokers or current smokers).

### 2.4. Clinical Measurements

Anthropometric measurements (weight, height, and waist circumference) and blood pressure were measured by well-trained staff. The anthropometric measurements were measured without shoes. Body weight was measured to the nearest 0.1 kg. Height and waist circumferences were measured to the nearest 0.5 cm. Height was measured with a stadiometer placing their heels against the rod and the head in Frankfort Plane position. Waist circumference was measured in standing position with a tape measure all around the body, at the level midway between the lower rib margin and the iliac crest. BMI was calculated as weight (kg) divided by height squared (m^2^). Systolic and diastolic blood pressures were measured 10 times during a period of 10 minutes, using an automated Dinamap Monitor (GE Healthcare, Freiburg, Germany). The average of the final three readings was used for each blood pressure parameter.

### 2.5. Biochemical Measurements

For analysis of lipids, glucose, uric acid, creatinine and the inflammation marker high sensitivity C-reactive protein (hs-CRP), blood samples were drawn in the morning between 8:00 and 10:00 am after a period of overnight fasting. Serum levels of total cholesterol and high-density lipoprotein (HDL) cholesterol were measured with an enzymatic colorimetric method, low-density lipoprotein (LDL) cholesterol with an enzymatic method and total triglycerides with a colorimetric UV method, all on a Roche Modular P chemistry analyzer (Roche, Basel, Switzerland). Fasting blood glucose was measured using a hexokinase method. HbA1c was determined in whole blood (EDTA-anticoagulated) by means of turbid metric inhibition immunoassay on a Cobas Integra 800 CTS analyzer (Roche Diagnostics Netherland BV, Almere, The Netherlands). Insulin resistance was measured by calculating the ratio total triglycerides/HDL cholesterol [27]. Serum uric acid and creatinine were measured on a Roche Modular P chemistry analyzer (Roche, Basel, Switzerland). The hs-CRP was determined by nephelometry (BN II system Siemens, Marburg, Germany).

### 2.6. Fatty Acid Analyses

EDTA-plasma samples were collected at baseline and stored at −80 °C until analyses of fatty acids were carried out. Analyses of fatty acids were performed at the Department of Laboratory Medicine of the University Medical Center Groningen, The Netherlands using the methodology as described by Hoving et al. [28]. In short, total lipids were extracted by the method of Folch et al., using 6 mL of chloroform-methanol (2:1) and a 200 µL EDTA-plasma sample [29]. Additionally, a shortened version of the method of Kaluzny et al. was used to isolate plasma cholesterol esters (CE), triglycerides (TG) and phospholipids (PL), using aminopropyl SPE columns for the separation (Isolute, Biotage) [30]. Fatty acids were transmethylated with methanolic-HCL into fatty acid methyl esters (FAME). The samples were extracted with hexane and eventually redissolved into 100 µL hexane. 100 µL of an internal standard for the quantification of fatty acids in CE (17:0) (50.1 mg/100 mL chloroform-methanol, 2:1 *v*/*v*), and 100 µL of an internal standard for the quantification of fatty acids in TG (19:0) (19.9 mg/100 mL chloroform-methanol, 2:1 *v*/*v*), both obtained from Sigma-Aldrich (Zwijndrecht, The Netherlands), were added before isolation of classes. For the quantification of fatty acids in PL, 100 µL of free fatty acid 19:0 (50.0 mg/100 mL methanol), obtained from Larodan (Solna, Sweden), was added after isolation of classes. 100 µL Butylated Hydroxytoluene (1 g/100 mL methanol) from Sigma-Aldrich (Zwijndrecht, The Netherlands) was added to prevent fatty acid oxidation.

Aliquots of 2 µL were injected into an Agilent model 6890 gas chromatography equipped with a 200 m × 0.25 mm polar column (CP Select for FAME) and detected with an Agilent 7683 series flame ionization detector. FAME were identified by comparing retention times with those of known standards (Supelco 37 component FAME mix (Sigma-Aldrich)). Fatty acids were calculated into mol%. Firstly, fatty acids were measured in a pilot of 96 samples to investigate whether the fatty acids of interest were detectable in plasma CE, TG and PL. Compared to plasma TG and PL, potentially interesting fatty acids were less detectable in plasma CE. Subsequently, we decided to only move forward with plasma TG and PL. The precision of the measurements was tested by calculating the variation coefficient from 10 replicate samples using quality-control samples (pooled plasma samples). The circulating fatty acids reported in this paper had a variation coefficient ≤15%.

### 2.7. Statistical Analyses

All analyses were performed using IBM SPSS Statistics, version 22.0 for Windows software (IBM, Armonk, NY, USA) and GraphPad Prism, version 5.03 for Windows software (Graphpad Software, La Jolla, CA, USA). At the start of the study, plasma circulating fatty acids were measured in 864 lifelines participants, whereof 776 had complete FFQ data. Cases with missing data either on circulating fatty acids or dietary intake were removed before analysis leaving 769 participants in the lifelines cohort with complete data. For illustrative purposes, baseline data are presented for the total population, and separately for males and females. Baseline data are presented as mean ± SD (normally distributed data), median (25th–75th percentile) (non-normally distributed data) or as number (%) (categorical data). Differences in baseline data between males and females were tested with an independent t-test (continuous data), Mann-Whitney U test (non-normally distributed data) or Chi-square test (categorical data). Multivariate linear regression analyses were carried out to investigate the association of circulating fatty acids in plasma TG and PL with dairy fat intake. Non-normal data were transformed before analyses. Models were adjusted for total energy intake (model 2), followed by further adjustments for age, education, smoking habits, BMI, total carbohydrates and total fat intake, fish fat intake, and total serum triglycerides and serum cholesterol (model 3–7). The same analyses were carried out to examine the association between circulating fatty acids and fish fat intake, except that the additional adjustments now included dairy fat intake instead of fish fat intake. As a sensitivity analysis, we also examined the association of EPA and DHA in plasma TG and PL with fish fat intake. To investigate our second purpose, multivariate linear regression analyses were used to examine the cross-sectional association of circulating fatty acids in plasma TG and PL with cardiovascular risk factors. Associations were adjusted for age and sex (model 2). In secondary analyses, models were additionally adjusted for education and smoking habits, total serum triglycerides, total serum cholesterol (only plasma PL), and fatty acids from de novo lipogenesis (C14:0 + C16:0 + C16:1 (*n*-7) from plasma TG). Lastly, the association between dairy fat intake and cardiovascular risk factors, and the association between fish fat intake and cardiovascular risk factors was investigated. Models were adjusted for total energy intake, age, sex, education, smoking habits, BMI, total carbohydrates and total fat intake, total fish fat intake/total dairy fat intake, and total serum triglycerides and serum cholesterol. All reported probability values are two-tailed, and a *p* ≤ 0.05 was considered statistically significant.

## 3. Results

### 3.1. The Lifelines Population

The characteristics of the lifelines population (total population and separately for males (*n* = 404) and females (*n* = 365)) can be found in Table 1. The total population had a mean age of 53.0 ± 15.5 years and a BMI of 26.0 ± 4.0 kg/m^2^. Differences in dietary intake were observed between males and females, i.e., the total energy intake, and the carbohydrate, protein and fat intake were significantly higher in males compared to females. However, after correction for individual energy intake (intake calculated as En%), only protein intake remained significantly higher in males compared to females (Table 1). Concerning intake of dairy fat and fish fat, if corrected for individual energy intake, dairy fat intake did not differ between males and females, while fish fat intake was higher in females compared to males (Table 1).

The circulating fatty acids C14:0, C15:0, C17:0, trans-C16:1 (*n*-7), trans-C18:1 (*n*-7) and CLA were measured in plasma TG and PL. Overall, the circulating fatty acids in plasma TG were higher compared to plasma PL. The quantitatively most abundant investigated marker was C14:0 (Total TG: 1.87 mol%, PL: 0.49 mol%), followed by C17:0 and C15:0 in both fractions.

### 3.2. Biomarkers for Dairy Fat or Fish Fat

**Figure 1:**
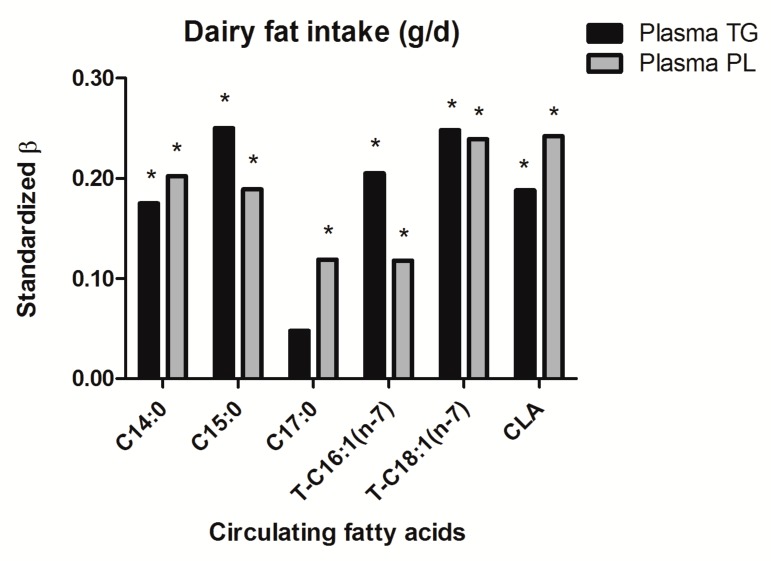
Association between circulating fatty acids from plasma triglycerides and phospholipids and dairy fat intake, adjusted for energy intake. * Equal or below significance level *p* ≤ 0.05. Abbreviations: β, beta; C14:0, Myristic acid; C15:0, Pentadecanoic acid; C17:0, Heptadecanoic acid; T-C16:1 (*n*-7), Trans-Palmitoleic acid; T-C18:1 (*n*-7), Vaccenic acid; CLA, Conjugated Linoleic acid; TG, Triglycerides; PL, Phospholipids.

To investigate whether the fatty acids in plasma TG and PL were dairy fat biomarkers, fish fat biomarkers or both, linear regression analyses were carried out. Firstly, the association with dairy fat intake was explored. After adjustment for energy intake, fatty acids in both plasma TG and PL, except C17:0 in plasma TG, were significantly associated with dairy fat intake (Figure 1). In plasma TG, C15:0 and trans-C18:1 (*n*-7) had the strongest association with dairy fat intake (both std.*β* = 0.25, *p* < 0.001). In plasma PL, trans-C18:1 (*n*-7) and CLA had the strongest association with dairy fat intake (both std.*β* = 0.24, *p* < 0.001), followed by C14:0 and C15:0 (std.*β* = 0.20, *p* < 0.001; std.*β* = 0.19, *p* < 0.001). Additional adjustments for age, sex, education, smoking habits, BMI, total carbohydrate and total fat intake, total fish fat intake, and total serum triglycerides and cholesterol did not alter the associations between circulating fatty acids and dairy fat intake (Table S1). If dairy fat intake was expressed as En%, associations with fatty acid biomarkers did not materially differ from associations found for dairy fat intake with adjustment for energy intake by means of linear regression.

**Figure 2:**
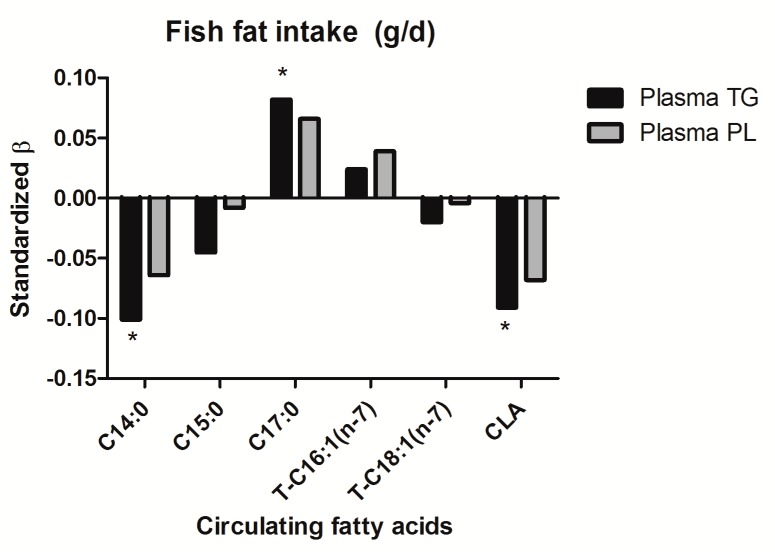
Association between circulating fatty acids from plasma triglycerides and phospholipids and fish fat intake, adjusted for energy intake. * Equal or below significance level *p* ≤ 0.05. Abbreviations: β, beta; C14:0, Myristic acid; C15:0, Pentadecanoic acid; C17:0, Heptadecanoic acid; T-C16:1 (*n*-7), Trans-Palmitoleic acid; T-C18:1 (*n*-7), Vaccenic acid; CLA, Conjugated Linoleic acid; TG, Triglycerides; PL, Phospholipids.

Secondly, the association with fish fat intake was explored. After adjustment for energy intake, only C14:0, C17:0 and CLA in plasma TG were significantly associated with fish fat intake (Figure 2). C14:0 and CLA were inversely associated (std.*β* = −0.10, *p* = 0.01; std.*β* = −0.09, *p* = 0.01, respectively), while C17:0 was positively associated with fish fat intake (std.*β* = 0.08, *p* = 0.03). The association with C14:0 and C17:0 disappeared after additional adjustments for age, sex, BMI, and total serum triglycerides and cholesterol (Model 7, Table S2). After adjustment for energy intake, fatty acids measured in plasma PL were not associated with fish fat intake. As a sensitivity analysis, the association of EPA and DHA with fish fat intake was explored as well. After adjustment for energy intake, age, sex, education and smoking habits, EPA and DHA in both plasma TG and PL were significantly associated with fish fat intake (plasma TG EPA: std.*β* = 0.27 and DHA: std.*β* = 0.45; plasma PL EPA: std.*β* = 0.25 and DHA: std.*β* = 0.46; *p* < 0.001) (Table S3). If fish fat intake was expressed as En%, associations with fatty acid biomarkers did not materially differ from associations found for fish fat intake with adjustment for energy intake by means of linear regression.

### 3.3. Circulating Fatty Acids in Plasma TG and Cardiovascular Risk Factors

The second aim of the study was to investigate whether the circulating fatty acids were associated with cardiovascular risk factors. Overall, C14:0 and C17:0 showed the strongest associations with cardiovascular risk factors (Table 2). C14:0 was positively associated with weight, waist circumference and BMI, while C15:0 and C17:0 showed an inverse association with weight, waist circumference and BMI. Additionally, C14:0 was positively associated with glucose and HbA1c% (std.*β* = 0.14, *p* < 0.001; std.*β* = 0.12, *p* < 0.01, respectively), while C17:0 was inversely associated with glucose (std.*β* = −0.13, *p* < 0.001). Furthermore, C15:0 and C17:0 were inversely associated with serum hs-CRP (std.*β* = −0.11, *p* < 0.05; std.*β* = −0.17, *p* < 0.01; respectively) and uric acid (std.*β* = −0.14, *p* < 0.05; std.*β* = −0.25, *p* < 0.001; respectively), and remained associated after adjustment for age and sex. Trans-C16:1 (*n*-7) and trans-C18:1 (*n*-7) became inversely associated with uric acid after adjustment for age and sex (std.*β* = −0.12, *p* < 0.05; std.*β* = −0.11; *p* < 0.05; respectively). C14:0 and C17:0 had a strong positive association with total triglycerides (std.*β* = 0.40, *p* < 0.001; std.*β* = −0.56, *p* < 0.001; respectively), and these associations remained after adjustment for age and sex. The age and sex adjusted analyses where therefore also adjusted for total triglycerides (Table S4). The association with weight, waist circumference and BMI remained for C15:0, but disappeared for C14:0 and C17:0. Additionally, the association with hs-CRP remained for C15:0 and C17:0, and the inverse association with uric acid remained for C15:0 and trans-C18:1 (*n*-7), but disappeared for C17:0, and became visible for C14:0. As an extra analyses, we adjusted the age and sex adjusted analyses for fatty acids from the *de* novo lipogenesis (DNL) instead of for total triglycerides. Interestingly, trans-C16:1 (*n*-7) and trans-C18:1 (*n*-7) became inversely associated with BMI (std.*β* = −0.11 and std.*β* = 0.10, both *p* < 0.05; respectively) and waist circumference (std.*β* = −0.101 and std.*β* = −0.07, both *p* < 0.05; respectively). 

### 3.4. Circulating Fatty Acids in Plasma PL and Cardiovascular Risk Factors

Besides fatty acids in plasma TG, the association between fatty acids from plasma PL and cardiovascular risk factors were also investigated. Overall, most associations were found with C17:0 and trans-C18:1 (*n*-7) (Table 3). C15:0, C17:0, trans-C16:1 (*n*-7) and trans-C18:1 (*n*-7) were inversely associated with weight and waist circumference. Additionally, C17:0 and trans-C18:1 (*n*-7) were inversely associated with BMI and glucose (std.*β* = −0.10, *p* < 0.05; std.*β* = −0.08, *p* < 0.05, respectively). Furthermore, C17:0 and trans-C18:1 (*n*-7) were inversely associated with hs-CRP (std.*β* = −0.22, *p* < 0.001; std.*β* = −0.16, *p* < 0.01, respectively), and all fatty acids, except for CLA, were inversely associated with uric acid, for which the strongest associations were found for C17:0 (std.*β* = 0.27, *p* < 0.001) and trans-C18:1 (*n*-7) (std.*β* = −0.24, *p* < 0.001). Associations remained after adjustments for age and sex.

C15:0, C17:0, trans-C16:1 (*n*-7) and trans-C18:1 (*n*-7) were inversely associated with total triglycerides (std.*β* = −0.16, *p* < 0.001; std.*β* = −0.24, *p* < 0.001; std.*β* = −0.15, *p* < 0.001; std.*β* = −0.10, *p* < 0.01, respectively), and these associations remained after adjustment for age and sex. To stay in line with the analyses carried out for plasma TG, the age and sex adjusted analyses were also adjusted for total triglycerides. Additionally, we adjusted the analyses for total cholesterol, since it is known that the fatty acids from PL are closely related to the fatty acids of plasma CE [31] (Table S5). The inverse association of C17:0 and Trans-C18:1 (*n*-7) with waist circumference and BMI remained. The association between CLA and BMI became inversely associated (std.*β* = −0.09, *p* < 0.05). C17:0 and trans-C18:1 (*n*-7) remained inversely associated with hs-CRP, while trans-C16:1 (*n*-7) became inversely associated with hs-CRP. Only C14:0, C17:0 and trans-C18:1 (*n*-7) remained inversely associated with uric acid. As an extra analyses, we adjusted the age and sex adjusted analyses for fatty acids from the DNL instead of for total triglycerides, but the associations did not change.

### 3.5. Dairy Fat Intake, Fish Fat Intake and Cardiovascular Risk Factors

The association of dairy fat, fish fat and cardiovascular risk factors was investigated as an extra analysis. Dairy fat intake was significantly associated with age (std.*β* = 0.36, *p* < 0.001) and sex (std.*β* = 0.08, *p* < 0.05) (Table S6). Furthermore, dairy fat intake was significantly associated with waist circumference, systolic and diastolic blood pressure, total, HDL and LDL cholesterol, glucose and HbA1c%. However, these associations disappeared after adjustment for age and sex. Dairy fat intake was inversely associated with uric acid, even after adjustment for age, sex, education, smoking habits, BMI, fish fat intake, and total carbohydrate and total fat intake. In addition to dairy fat intake, fish fat intake was also significantly associated with age (std.*β* = 0.08, *p* ≤ 0.05), but not with sex (Table S7). Furthermore, fish fat intake was positively associated with HDL cholesterol (std.*β* = 0.10, *p* < 0.01), and inversely associated with total triglycerides (std.*β* = −0.12, *p* < 0.01) and the TG/HDL-c ratio. The associations remained after adjustments for age, sex, education, smoking habits, BMI, total dairy fat intake, total carbohydrate and total fat intake.

## 4. Discussion

The current paper showed that C14:0, C15:0, C17:0, trans-C16:1 (*n*-7), trans-C18:1 (*n*-7) and CLA were solid biomarkers to predict dairy fat intake in the general population, while in contrast, C15:0 and C17:0 were not confirmed as strong biomarkers for fish fat intake. The dairy fatty acids C15:0 and C17:0 in plasma TG and C15:0, C17:0 and trans-C18:1 (*n*-7) in plasma PL had an inverse association with cardiovascular risk factors such as weight, waist circumference and BMI. Additionally, dairy fat biomarkers were also inversely associated with diabetes-related outcomes, the inflammatory marker hs-CRP and the kidney marker uric acid.

For many years now the saturated fatty acids C15:0 and C17:0, and to a lesser extend C14:0 have been described as biomarkers to predict dairy and dairy fat intake [5,12,15,32,33,34,35,36]. Additionally, the trans fatty acids trans-C16:1 (*n*-7), trans-C18:1 (*n*-7) and CLA were added to the list of biomarkers [5,15,36,37,38]. In line with the previous studies, we also found that the current mentioned biomarkers are predictors of dairy fat intake in the general population, of which the strongest associations were found with TG C15:0, TG trans-C18:1 (*n*-7), PL trans-C18:1 (*n*-7) and PL CLA. Associations of dairy fat intake with trans-C18:1 (*n*-7) and CLA have hardly been explored. One study investigated the association between dairy fat intake and erythrocyte trans-C18:1 (*n*-7), but found no significant association [36]. Furthermore, associations between CLA and dairy fat intake are more commonly investigated in adipose tissue. A case-control study that investigated the association between CLA and the risk of myocardial infarction demonstrated that CLA was significantly associated with dairy intake in the Costa Rican population (*r* = 0.31) [37]. In contrast to trans-C18:1 (*n*-7) and CLA, the association between dairy fat intake and plasma C15:0 has been investigated and confirmed in several studies [12,13,14,15]. In a cross-sectional study of Lund-Blix et al., it was for example found that plasma PL C15:0 and total dairy fat were significantly associated with an *r* of 0.39 [14].

Besides dairy fat, the saturated fatty acids C15:0 and C17:0 have also been described as potential fish fat biomarkers. The fatty acids can be found in marine water fish and have recently found to be related to DHA [18,19]. C15:0 and C17:0 were therefore presumed to be invalid dairy fat biomarkers in a population with a high fish consumption. Participants from the current study showed a low intake of fish (11.7 g/day) and a high intake of dairy (322 g/day). Subsequently, C15:0 and C17:0, and the other dairy fat biomarkers were not confirmed as strong biomarkers for fish fat intake. C15:0, C17:0 and the other circulating fatty acids are therefore valid dairy fat biomarkers for the general population in the northern part of the Netherlands.

In the current study, significant associations were found between dairy fat biomarkers and cardiovascular risk factors. The association between the circulating dairy fat biomarkers C14:0, C15:0, C17:0 and trans-C16:1 (*n*-7) and cardiovascular health has been investigated before and an illustrative overview of papers investigating this association can be found in Table S8. One of the main interesting outcomes of the current paper is the inverse association of the saturated fatty acids C15:0 and C17:0 with weight, waist circumference and BMI. A Swedish prospective study regarding risk factors for ischemic heart disease in 62 70-year old men found data that was in line with the current findings. Researchers showed an inverse association between plasma CE C15:0 and weight (*r*^2^ = −0.36), waist circumference (*r*^2^ = −0.28) and BMI (*r*^2^ = −0.39) [32]. A more recent Swedish study on the effect of dairy fat biomarkers and the risk to develop myocardial infarction in an adult population demonstrated an inverse association of plasma PL C15:0, PL C17:0 and PL C15:0 + C17:0 with BMI (*r* = −0.08, *r* = −0.14 and *r* = −0.14 respectively) [6]. Additionally, serum PL C15:0 and C17:0 were inversely associated with abdominal obesity (*r* = −0.22 and *r* = −0.30, respectively) as was found in a cross-sectional study with 301 healthy 63-year old men [39]. The inverse association between the saturated fatty acids and adiposity markers are in line with a recent published paper on dairy and overweight [40]. Moreover, the authors used data from the Lifelines Cohort also. However, while we investigated the association between dairy fat biomarkers and adiposity, they investigated the association between dairy fat products and adiposity. The investigators found an inverse association between full-fat dairy product intake and BMI-defined overweight (≥25–30 kg/m^2^) and obesity (≥30 kg/m^2^). Additionally, age and sex adjusted multivariate linear regression analyses identified inverse associations of full-fat dairy product intake (per 100g) with waist circumference (β = −0.39) and BMI (β = −0.23). In line with this paper, a systematic review of observational studies on the relationship between dairy intake and obesity, cardiovascular, and metabolic disease found an inverse association between high fat dairy consumption and measurements of adiposity in 11 out of 16 studies [41].

The ruminant derived trans fatty acids were also found to be related to weight, waist circumference and BMI in the Lifelines Cohort. The plasma PL trans fatty acids trans-C16:1 (*n*-7) and trans-C18:1 (*n*-7) were inversely related to weight and waist circumference. Additionally, trans-C18:1 (*n*-7) was also inversely associated with BMI. In secondary analyses, plasma TG trans fatty acids trans-C16:1 (*n*-7) and trans-C18:1 (*n*-7) became inversely associated with waist circumference and BMI after adjustment for the fatty acids from DNL. The cardiovascular health study, a prospective cohort study from the U.S. including 3736 adult participants investigated the association between trans-C16:1 (*n*-7) and metabolic risk and diabetes type 2 [42]. The researchers found a significant reduction of 1.8% (across quintiles) in BMI and waist circumference. Furthermore, a study in which the intake of ruminant derived trans fat was measured by FFQs also suggested a favorable effect on cardiovascular health. Results from this cohort showed an inverse association between intake of ruminant derived trans fat and changes in weight [43].

Concerning the effect on diabetes-related outcomes, plasma TG C14:0 was positively associated with glucose and HbA1c%, however this association did not remain after adjustment for total serum triglycerides. Plasma TG C17:0, PL C17:0 and PL trans-C18:1 (*n*-7) were inversely associated with glucose. Additionally, trans-C18:1 (*n*-7) was inversely associated with HbA1c%. All other dairy fat biomarkers were not associated with the diabetes-related outcomes. In a cross-sectional study with 17 men and women without nonalcoholic fatty liver disease, it was found that serum PL C15:0, C17:0 and trans-C16:1 (*n*-7), and free fatty acid C15:0 and C17:0 were inversely related to fasting plasma glucose, the area under the curve for glucose during an oral glucose tolerance test (OGTT) and liver fat [44]. Additionally, a prospective, case-control study with Swedish adults demonstrated that serum PL C17:0 and C15:0 + C17:0, but not C15:0, were inversely associated with fasting glucose (*r* = −0.13 and *r* = −0.16, respectively) [6]. Moreover, a cross-sectional study with 795 elderly men showed an inverse association between adipose tissue C17:0 and insulin sensitivity, but not between adipose tissue C15:0 and insulin sensitivity [45]. While data from the Lifelines Cohort demonstrated an inverse association between dairy fat biomarkers and diabetes-related outcomes, an opposite association of high-fat dairy product intake with pre-diabetes and newly diagnosed type 2 diabetes was actually found in the same cohort [46]. The authors suggested that the positive association between dairy fat intake and diabetes-related outcomes may potentially not depend on the ‘fat content’ of a dairy product, but to the individual dairy product intake.

Little is known about the mechanism behind the inverse association between dairy fat biomarkers and cardiovascular risk factors such as weight, waist circumference and BMI. One potential mechanism by which dairy fat may exert beneficial effects on cardiovascular risk factors, may be via reducing chronic inflammation and lipid peroxidation [41,47]. This mechanism was suggested by a cross-sectional study among 305 adolescents, in which it was demonstrated that serum C15:0 and C17:0 were inversely associated with inflammation markers and oxidative stress in the overweight group [47]. The inverse association between dairy fat biomarkers and inflammation was confirmed in the current study, and in earlier published papers [47,48]. While it is commonly known that inflammatory markers and oxidative stress are positively related with cardiovascular risk factors [47,49,50,51], dairy fat biomarkers may actually reduce inflammatory markers, and subsequently cardiovascular risk factors.

In addition to the potential mechanism ascribed above, uric acid may also be involved in this process. Uric acid was found to be inversely related with dairy fat biomarkers in plasma TG and PL. The strongest associations were found with plasma PL C17:0 and PL trans-C18:1(*n*-7). Inverse associations between these dairy fat biomarkers and uric acid remained after adjustment for age, sex, education, smoking habits, total serum triglycerides and serum cholesterol. In addition to the dairy fat biomarkers, there was also an inverse association observed between dairy fat intake measured by FFQ, and uric acid and has been observed in the literature before [52]. While an increased uric acid level may increase the risk for the development of cardiovascular diseases [53,54], perhaps by activating a complex mechanism involving inflammatory and oxidative related mechanisms [55,56], it can be hypothesized that the opposite may actually reduce the risk for the development of cardiovascular risk. Overall, more research warrants an investigation of the potential mechanism between dairy fat biomarkers, uric acid levels, inflammation and cardiovascular health.

Interestingly, the associations between circulating fatty acids and cardiovascular risk factors were less strong in plasma TG compared to plasma PL. This might probably be due to the fact that the association between plasma TG trans fatty acids and cardiovascular risk factors can be influenced by recent dietary intakes and de novo lipogenesis, whereas this is less for plasma PL [57]. In our cohort, the association between fatty acids from de novo lipogenesis (i.e., C14:0, C16:0, C16:1 (*n*-7)) measured in plasma TG) were significantly associated with total triglycerides. Additionally, adjusting for the fatty acids from de novo lipogenesis changed associations between circulating dairy fat biomarkers and cardiovascular risk factors. Our results therefore also suggest that it is preferable to measure fatty acids in phospholipids to investigate associations with cardiovascular risk factors.

A main strength of this paper is the inclusion of the relatively large amount of participants. Furthermore, the association between circulating fatty acids and cardiovascular risk factors were investigated. Lifelines is building a biobank and database and for this purpose, Lifelines has collected and is still collecting information via questionnaires, physical examinations and biological samples, including information on several cardiovascular risk factors. This huge collection of data and samples allowed us to investigate a brought range of cardiovascular health aspects in relation to dairy fat biomarkers, dairy fat intake and fish fat intake. A third strong point is that we measured the fatty acids in two different compartments, i.e., plasma TG and PL, providing the opportunity to investigate whether the association of dairy fat biomarkers with cardiovascular risk factors were consistent in plasma lipid classes with widely differing functions and half-lives.

Some limitations of this study should be addressed. First, given the observational nature of this study, it is impossible to draw a definite conclusion about the causality of the association of the circulating fatty acids with dairy fat, fish fat and cardiovascular risk factors. Secondly, the Lifelines cohort study uses an FFQ to measure dietary intake of the participants which is a method based on self-report, and therefore subject for recall bias. It is possible that the intakes of dairy and fish fat are over- or underreported. However, since there is no gold standard for measuring dietary intakes so far, FFQs are still seen as one of the best methods since they are able to capture usual, individual, long-term dietary intakes. Additionally, the burden for the participant is low [58,59]. Thirdly, we used Hoving’s method for the fatty acid analyses. The coefficients of variation for measuring circulating fatty acids in plasma was relatively high. Despite this limitation, we still found several associations between circulating fatty acids and cardiovascular risk factors. Potentially, using a method that allows for measurement of circulating fatty acids with lower coefficients of variation, such as Glaser’s method, might have given stronger associations between circulating fatty acids and cardiovascular risk factors [60].

## 5. Conclusions

This paper confirmed that the current known dairy fatty acid biomarkers are solid biomarkers to predict dairy fat intake in the general population of the Netherlands. Additionally, the fat biomarkers were inversely associated with adiposity, diabetes and inflammatory outcomes, suggesting that dairy fat intake may have a beneficial effect on cardiovascular health. Longitudinal studies are needed to further investigate the influence of dairy fat intake on cardiovascular risk and overall health.

## Figures and Tables

**Table 1:** Characteristics of the total Lifelines Population, separate for males and females.

Baseline Characteristics	Total *N* = 769	Males *N* = 404	Females *N* = 365	*p*-value
General				
Age visit 1, years	53.0 ± 15.5	53.3 ± 15.2	52.7 ± 16.0	0.60
Weight, kg	79.3 ± 14.2	85.7 ± 11.9	72.3 ± 13.2	<0.001
BMI, kg/m^2^	26.0 ± 4.0	26.3 ± 3.4	25.8 ± 4.6	0.08
Smoking, yes (%)	115 (15.0%)	68 (16.8%)	47 (12.9%)	0.13
SBP, mmHg	126.5 ± 17.0	129.5 ± 15.1	123.1 ± 18.2	<0.001
DBP, mmHg	73.7 ± 9.7	76.6 ± 9.8	70.5 ± 8.4	<0.001
Laboratory measurements				
Total Cholesterol, mmol/L	5.2 ± 1.0	5.1 ± 1.0	5.3 ± 1.1	0.03
HDL cholesterol, mmol/L	1.5 ± 0.4	1.3 ± 0.3	1.7 ± 0.4	<0.001
LDL cholesterol, mmol/L	3.3 ± 0.9	3.3 ± 0.9	3.3 ± 1.0	0.27
Total Triglycerides, mmol/L	1.0 (0.8–1.5)	1.2 (0.9–1.6)	1.0 (0.7–1.3)	<0.001
Total TG/HDL-c ratio	0.72 (0.45–1.17)	0.92 (0.60–1.43)	0.56 (0.38–0.88)	<0.001
Glucose, mmol/L	5.1 ± 0.7	5.2 ± 0.6	5.0 ± 0.7	0.003
HbA1c, %	5.6 ± 0.4	5.6 ± 0.4	5.6 ± 0.4	0.99
Creatinine, umol/L	76.0 ± 13.6	83.6 ± 12.3	67.6 ± 9.3	<0.001
Hs-CRP, mg/L ^1^	1.0 (0.5–2.2)	0.9 (0.4–1.9)	1.1 (0.5–2.9)	0.05
Uric acid, mmol/L ^1^	0.31 ± 0.1	0.34 ± 0.1	0.28 ± 0.1	<0.001
Dietary intake (FFQ)				
Energy intake, Kcal/day	1971 ± 624	2206 ± 665	1711 ± 449	<0.001
Total carbohydrates, g/day	228.8 ± 76.7	254.4 ± 80.7	200.4 ± 60.6	<0.001
Total carbohydrates, En%	46.4 ± 6.0	46.2 ± 5.7	46.7 ± 6.2	0.08
Total protein, g/day	74.8 ± 20.9	81.5 ± 22.6	67.3 ± 15.8	<0.001
Total protein, En%	15.5 ± 2.4	15.0 ± 2.1	16.0 ± 2.6	0.05
Total fat, g/day	77.2 ± 30.0	87.1 ± 32.7	66.2 ± 22.0	<0.001
Total fat, En%	34.9 ± 5.1	35.1 ± 5.0	34.6 ± 5.1	0.82
Total Dairy intake, g/day	322 (209–447)	315 (202–444)	324 (216–447)	0.99
Total Dairy fat intake, g/day	12.3 (8.4–17.4)	13.5 (9.0–18.3)	11.3 (8.0–15.6)	<0.001
Total Dairy fat intake (En%)	6.0 (4.3–7.8)	5.7 (3.4–7.4)	6.2 (4.5–8.1)	0.90
Total Fish intake, g/day	11.7 (5.6–17.5)	12.0 (6.8–17.4)	11.6 (5.2–17.5)	0.87
Total Fish fat intake, g/day	1.14 (0.53–1.75)	1.16 (0.58–1.74)	1.10 (0.48–1.77)	0.50
Total Fish fat intake (En%)	0.52 (0.24–0.88)	0.49 (0.23–0.77)	0.57 (0.26–1.01)	0.01
Fatty acid status in plasma TG				
Myristic acid, mol%	1.87 (1.40–2.48)	1.98 (1.49–2.68)	1.74 (1.35–2.35)	<0.001
Pentadecanoic acid, mol%	0.30 (0.25–0.35)	0.29 (0.24–0.35)	0.30 (0.25–0.35)	0.27
Heptadecanoic acid, mol%	0.49 (0.41–0.59)	0.48 (0.39–0.58)	0.52 (0.44–0.62)	<0.001
Trans-Palmitoleic acid, mol%	0.03 (0.02–0.04)	0.03 (0.02–0.04)	0.03 (0.02–0.04)	0.05
Vaccenic acid, mol%	0.10 (0.06–0.14)	0.10 (0.07–0.15)	0.10 (0.07–0.14)	0.30
Conjugated Linoleic acid, mol%	0.08 (0.05–0.10)	0.07 (0.05–0.10)	0.07 (0.05–0.10)	0.24
Fatty acid status in plasma PL				
Myristic acid, mol%	0.49 (0.41–0.57)	0.47 (0.40–0.55)	0.50 (0.43–0.58)	<0.001
Pentadecanoic acid, mol%	0.28 (0.24–0.33)	0.28 (0.24–0.33)	0.29 (0.25–0.33)	0.09
Heptadecanoic acid, mol%	0.40 (0.36–0.44)	0.39 (0.35–0.44)	0.40 (0.36–0.45)	0.08
Trans-Palmitoleic acid, mol%	0.02 (0.01–0.03)	0.02 (0.01–0.02)	0.02 (0.02–0.03)	0.002
Vaccenic acid, mol%	0.09 (0.07–0.12)	0.09 (0.06–0.11)	0.09 (0.07–0.12)	0.002
Conjugated Linoleic acid, mol%	0.03 (0.02–0.04)	0.03 (0.02–0.04)	0.03 (0.02–0.04)	0.01

Data are presented as mean ± SD, median (25–75th percentile) or number (%). Differences between gender were tested by independent *t*-test, Mann-Whitney U or Chi-square test. Abbreviations: SD, standard deviation;; BMI, body mass index; SBP, systolic blood pressure; DBP, diastolic blood pressure; TG, Triglycerides; TG/HDL-c ratio, Total Triglycerides/HDL cholesterol ratio; HbA1C, Hemoglobin A1C; hs-CRP, High sensitivity C-reactive protein; FFQ, Food Frequency Questionnaire; Kcal, Kilocalories; PL, Phospholipids. ^1.^ Hs-Crp was available in *n* = 412, uric acid was available in *n* = 348.

**Table 2:** Association between circulating fatty acid markers from plasma triglycerides (TG) and cardiovascular risk factors.

Dependent Variable	C14:0		C15:0		C17:0		Trans-C16:1 (*n*-7)		Trans-C18:1 (*n*-7)		CLA	
	Model 1	Model 2	Model 1	Model 2	Model 1	Model 2	Model 1	Model 2	Model 1	Model 2	Model 1	Model 2
Age (years)	0.063		0.071 *		−0.027		0.022		0.088 *		0.047	
Sex	−0.129 ***		0.023		0.152 ***		0.066		−0.058		0.037	
Weight (kg)	0.132 ***	0.070 *	−0.091 *	−0.083 *	−0.187 ***	−0.117 ***	−0.073	−0.043	0.030	0.000	−0.024	−0.008
Waist circumference	0.146 ***	0.084 *	−0.096 *	−0.112 ***	−0.201 ***	−0.144 ***	−0.076	−0.061	0.048	0.000	−0.020	−0.022
BMI (kg/m^2^)	0.091 *	0.071 *	−0.117 **	−0.132 ***	−0.181 ***	−0.170 ***	−0.073	−0.075	−0.008	−0.031	−0.022	−0.030
SBP (mmHg)	0.075 *	0.024	−0.042	−0.069 *	−0.103 *	−0.064 *	−0.049	−0.047	0.019	−0.031	−0.043	−0.057
DBP (mmHg)	0.128 ***	0.068 *	−0.053	−0.070 *	−0.147 ***	−0.093 *	−0.028	−0.015	0.054	0.005	0.014	0.010
Total C (mmol/L)	0.152 ***	0.147 ***	0.000	−0.024	−0.296 ***	−0.308 ***	0.051	0.039	0.128 ***	0.108 **	0.079 *	0.062
HDL-c (mmol/L)	−0.262 ***	−0.213 ***	−0.035	−0.052	0.220 ***	0.157 ***	0.051	0.019	−0.060	−0.041	−0.057	−0.078 *
LDL-c (mmol/L)	0.172 ***	0.154 ***	0.017	−0.001	−0.277 ***	−0.270 ***	0.029	0.025	0.119 **	0.095 *	0.070 *	0.059
Total TG	0.401 ***	0.370 ***	−0.012	−0.018	−0.563 ***	−0.537 ***	−0.077	−0.066	0.127 ***	0.103 **	0.124 **	0.126 ***
TG/HDL-c ratio	0.396 ***	0.354 ***	0.003	0.005	−0.503 ***	−0.459 ***	−0.076	−0.055	0.120 **	0.095 **	0.114 **	0.123 ***
Glucose (mmol/L)	0.136 ***	0.104 **	−0.031	−0.054	−0.128 **	−0.105 **	−0.031	−0.033	0.063	0.026	0.019	0.006
HbA1c (%)	0.119**	0.088 *	−0.002	−0.041	−0.040	−0.027	−0.018	−0.031	0.041	−0.006	−0.014	−0.040
Hs-CRP (mg/L) ^1^	0.021	0.039	−0.109 *	−0.118 *	−0.168 **	−0.203 ***	−0.057	−0.076	−0.063	−0.057	−0.019	−0.027
Creatinine (umol/L)	0.100 *	0.016	−0.029	−0.025	−0.147 ***	−0.055	−0.016	0.020	0.052	0.005	−0.011	0.005
Uric acid (mmol/L) ^1^	0.131 *	0.082	−0.139 *	−0.160 ***	−0.249 ***	−0.233 ***	−0.096	−0.123 *	−0.087	−0.108 *	−0.042	−0.038
Ureum (mmol/L) ^1^	0.031	0.007	0.146 **	0.122 *	0.090	0.086	0.113	0.096	0.150 **	0.137 **	0.105 *	0.080

**Model 1:** Crude model; **Model 2:** Model 1 + additional adjustments for age and sex. Dependent: cardiovascular risk factors, independent: circulating fatty acids. Associations between fatty acid status and cardiovascular risk factor are reported as standardized β’s. *p*-value: * = ≤ 0.05, ** = < 0.01, *** = < 0.001. The significant values are highlighted in bold. Abbreviations: BMI, body mass index; SBP, systolic blood pressure; DBP, diastolic blood pressure; Total C; total cholesterol; HDL-c, high density lipoprotein cholesterol; LDL-c, low density lipoprotein cholesterol; Total TG, total triglycerides; TG/HDL-c ratio, total triglycerides/HDL cholesterol; HbA1c, Hemoglobin A1C; Hs-CRP, High sensitivity C-reactive protein. ^1^ Hs-Crp data was available in *n* = 412, Uric acid and ureum data were available in *n* = 348.

**Table 3:** Association between circulating fatty acid markers from plasma Phospholipids (PL) and cardiovascular risk factors.

Dependent Variable	C14:0		C15:0		C17:0		Trans-C16:1 (*n*-7)		Trans-C18:1 (*n*-7)		CLA	
	Model 1	Model 2	Model 1	Model 2	Model 1	Model 2	Model 1	Model 2	Model 1	Model 2	Model 1	Model 2
Age (years)	0.071 *		0.070 *		0.078 *		0.008		0.071 *		0.061	
Sex	0.141 ***		0.066		0.063		0.110 **		0.102 *		0.103 **	
Weight (kg)	−0.071 *	−0.008	−0.084 *	−0.056	−0.173 ***	−0.148 ***	−0.071 *	−0.020	−0.114 **	−0.069 *	−0.046	0.000
Waist circumference	−0.037	−0.012	−0.092 *	−0.094 **	−0.176 ***	−0.182 ***	−0.102 **	−0.068 *	−0.102 *	−0.092 **	−0.040	−0.025
BMI (kg/m^2^)	−0.022	−0.030	−0.057	−0.069 *	−0.170 ***	−0.185 ***	−0.069	−0.065	−0.107 **	−0.119 **	−0.037	−0.044
SBP (mmHg)	−0.029	−0.036	−0.024	−0.044	−0.045	−0.069 *	−0.078 *	−0.062 *	−0.081 *	−0.095 **	0.055	−0.064 *
DBP (mmHg)	−0.029	−0.010	−0.063	−0.068 *	−0.121 **	−0.130 ***	−0.075 *	−0.044	−0.072 *	−0.066 *	−0.003	0.008
Total C (mmol/L)	0.153 ***	0.123 ***	−0.058	−0.085 *	−0.212 ***	−0.243 ***	−0.034	−0.047	0.047	0.017	0.173 ***	0.148 ***
HDL-c (mmol/L)	0.068	−0.001	0.072 *	0.036	−0.015	−0.050	0.053	0.003	0.093 *	0.042	0.084 *	0.033
LDL-c (mmol/L)	0.137 ***	0.127 ***	−0.054	−0.071 *	−0.157 ***	−0.178 ***	−0.022	−0.021	0.033	0.018	0.121 **	0.110 **
Total TG	0.018	0.040	−0.155 ***	−0.153 ***	−0.235 ***	−0.235 ***	−0.151 ***	−0.130 ***	−0.098 *	−0.088 *	0.091 *	0.106 **
TG/HDL-c ratio	−0.015	0.028	−0.142 ***	−0.127 ***	−0.169 ***	−0.135 ***	−0.131 ***	−0.095 **	−0.108 **	−0.080 *	0.033	0.064
Glucose (mmol/L)	0.033	0.025	−0.048	−0.065	−0.100 *	−0.121 ***	−0.035	−0.027	−0.077 *	−0.095 *	−0.036	−0.048
HbA1c (%)	0.014	−0.027	−0.001	−0.040	−0.008	−0.052	−0.023	−0.029	−0.057	−0.099 **	0.006	−0.029
Hs-CRP (mg/L) ^1^	−0.030	−0.055	−0.074	−0.087	−0.223 ***	−0.233 ***	−0.089	−0.117 *	−0.160 **	−0.179 ***	0.023	0.005
Creatinine (umol/L)	−0.112 **	−0.040	−0.029	0.000	−0.107 **	−0.082 *	−0.059	0.005	−0.093 *	−0.044	−0.062	−0.010
Uric acid (mmol/L) ^1^	−0.140 *	−0.096	−0.157 **	−0.130 *	−0.270 ***	−0.258 ***	−0.140 *	−0.131 *	−0.243 ***	−0.208 ***	−0.029	−0.065
Ureum (mmol/L) ^1^	0.021	0.007	0.188 ***	0.186	0.109 *	0.089	0.097	0.096	0.105 *	0.099	0.032	0.026

**Model 1:** Crude model; **Model 2:** Model 1 + additional adjustments for age and sex. Dependent: cardiovascular risk factors, independent: circulating fatty acids. Associations between fatty acid status and cardiovascular risk factor are reported as standardized β’s. *p*-value: * = ≤ 0.05, ** = < 0.01, *** = < 0.001. The significant values are highlighted in bold. Abbreviations: BMI, body mass index; SBP, systolic blood pressure; DBP, diastolic blood pressure; Total C; total cholesterol; HDL-c, high density lipoprotein cholesterol; LDL-c, low density lipoprotein cholesterol; Total TG, total triglycerides; TG/HDL-c ratio, total triglycerides/HDL cholesterol; HbA1c, Hemoglobin A1C; Hs-CRP, High sensitivity C-reactive protein. ^1.^ Hs-Crp data was available in *n* = 412, Uric acid and ureum data were available in *n* = 348.

## Supplementary Materials

The following are available online at https://www.mdpi.com/2072-6643/11/5/1099/s1, Table S1: Cross-sectional association between circulating fatty acids and dairy fat intake; Table S2: Cross-sectional association between circulating fatty acids and fish fat intake; Table S3: Association between the circulating fatty acids EPA and DHA and fish fat intake; Table S4: Association between fatty acid markers from plasma triglycerides (TG) and cardiovascular risk factors.; Table S5: Association between fatty acid markers from plasma phospholipids (PL) and cardiovascular risk factors.; Table S6: Association between dairy fat intake and cardiovascular risk factors; Table S7: Association between fish fat intake and cardiovascular risk factors; Table S8A–C: Papers investigating the association between dairy fat biomarkers and cardiovascular health (based on an illustrative search).

## References

[1] WHO (World Health Organization) (2017). Cardiovascular Diseases (CVDs) Fact Sheet.

[2] Wanders A.J., Alssema M., de Koning E.J., le Cessie S., de Vries J.H., Zock P.L., Rosendaal F.R., Heijer M.D., de Mutsert R. (2017). Fatty Acid Intake and its Dietary Sources in Relation with Markers of Type 2 Diabetes Risk: The NEO Study. Eur. J. Clin. Nutr..

[3] De Oliveira Otto M.C., Mozaffarian D., Kromhout D., Bertoni A.G., Sibley C.T., Jacobs D.R., Nettleton J.A. (2012). Dietary Intake of Saturated Fat by Food Source and Incident Cardiovascular Disease: The Multi-Ethnic Study of Atherosclerosis. Am. J. Clin. Nutr..

[4] De Oliveira Otto M.C., Nettleton J.A., Lemaitre R.N., Steffen L.M., Kromhout D., Rich S.S., Tsai M.Y., Jacobs D.R., Mozaffarian D. (2013). Biomarkers of Dairy Fatty Acids and Risk of Cardiovascular Disease in the Multi-Ethnic Study of Atherosclerosis. J. Am. Heart Assoc..

[5] Sun Q., Ma J., Campos H., Hu F.B. (2007). Plasma and Erythrocyte Biomarkers of Dairy Fat Intake and Risk of Ischemic Heart Disease. Am. J. Clin. Nutr..

[6] Warensjo E., Jansson J.H., Cederholm T., Boman K., Eliasson M., Hallmans G., Johansson I., Sjogren P. (2010). Biomarkers of Milk Fat and the Risk of Myocardial Infarction in Men and Women: A Prospective, Matched Case-Control Study. Am. J. Clin. Nutr..

[7] Warensjo E., Smedman A., Stegmayr B., Hallmans G., Weinehall L., Vessby B., Johansson I. (2009). Stroke and Plasma Markers of Milk Fat Intake—A Prospective Nested Case-Control Study. Nutr. J..

[8] Gebauer S.K., Chardigny J.M., Jakobsen M.U., Lamarche B., Lock A.L., Proctor S.D., Baer D.J. (2011). Effects of Ruminant Trans Fatty Acids on Cardiovascular Disease and Cancer: A Comprehensive Review of Epidemiological, Clinical, and Mechanistic Studies. Adv. Nutr..

[9] Kromhout D., Menotti A., Bloemberg B., Aravanis C., Blackburn H., Buzina R., Dontas A.S., Fidanza F., Giampaoli S., Jansen A. (1995). Dietary Saturated and Trans Fatty Acids and Cholesterol and 25-Year Mortality from Coronary Heart Disease: The Seven Countries Study. Prev. Med..

[10] Li H., Zhang Q., Song J., Wang A., Zou Y., Ding L., Wen Y. (2017). Plasma Trans-Fatty Acids Levels and Mortality: A Cohort Study Based on 1999–2000 National Health and Nutrition Examination Survey (NHANES). Lipids Health Dis..

[11] Mozaffarian D., Katan M.B., Ascherio A., Stampfer M.J., Willett W.C. (2006). Trans Fatty Acids and Cardiovascular Disease. N. Engl. J. Med..

[12] Sofie Biong A., Berstad P., Pedersen J.I. (2006). Biomarkers for Intake of Dairy Fat and Dairy Products. Eur. J. Lipid Sci. Technol..

[13] Warensjo Lemming E., Nalsen C., Becker W., Ridefelt P., Mattisson I., Lindroos A.K. (2015). Relative Validation of the Dietary Intake of Fatty Acids among Adults in the Swedish National Dietary Survey using Plasma Phospholipid Fatty Acid Composition. J. Nutr. Sci..

[14] Lund-Blix N.A., Ronningen K.S., Boas H., Tapia G., Andersen L.F. (2016). Plasma Phospholipid Pentadecanoic Acid, EPA, and DHA, and the Frequency of Dairy and Fish Product Intake in Young Children. Food Nutr. Res..

[15] Yakoob M.Y., Shi P., Willett W.C., Rexrode K.M., Campos H., Orav E.J., Hu F.B., Mozaffarian D. (2016). Circulating Biomarkers of Dairy Fat and Risk of Incident Diabetes Mellitus among Men and Women in the United States in Two Large Prospective Cohorts. Circulation.

[16] Aslibekyan S., Campos H., Baylin A. (2012). Biomarkers of Dairy Intake and the Risk of Heart Disease. Nutr. Metab. Cardiovasc. Dis..

[17] Ratnayake W.M. (2015). Concerns about the use of 15:0, 17:0, and Trans-16:1n-7 as Biomarkers of Dairy Fat Intake in Recent Observational Studies that Suggest Beneficial Effects of Dairy Food on Incidence of Diabetes and Stroke. Am. J. Clin. Nutr..

[18] Ozogul Y., Ozogul F., Cicek E., Polat A., Kuley E. (2009). Fat Content and Fatty Acid Compositions of 34 Marine Water Fish Species from the Mediterranean Sea. Int. J. Food Sci. Nutr..

[19] Lankinen M., Schwab U. (2015). Biomarkers of Dairy Fat. Am. J. Clin. Nutr..

[20] Mori T.A. (2017). Marine OMEGA-3 Fatty Acids in the Prevention of Cardiovascular Disease. Fitoterapia.

[21] Whelton S.P., He J., Whelton P.K., Muntner P. (2004). Meta-Analysis of Observational Studies on Fish Intake and Coronary Heart Disease. Am. J. Cardiol..

[22] Maki K.C., Palacios O.M., Bell M., Toth P.P. (2017). Use of Supplemental Long-Chain Omega-3 Fatty Acids and Risk for Cardiac Death: An Updated Meta-Analysis and Review of Research Gaps. J. Clin. Lipidol..

[23] Stolk R.P., Rosmalen J.G., Postma D.S., de Boer R.A., Navis G., Slaets J.P., Ormel J., Wolffenbuttel B.H. (2008). Universal Risk Factors for Multifactorial Diseases: LifeLines: A Three-Generation Population-Based Study. Eur. J. Epidemiol..

[24] Scholtens S., Smidt N., Swertz M.A., Bakker S.J., Dotinga A., Vonk J.M., van Dijk F., van Zon S.K., Wijmenga C., Wolffenbuttel B.H. (2015). Cohort Profile: LifeLines, a Three-Generation Cohort Study and Biobank. Int. J. Epidemiol..

[25] Klijs B., Scholtens S., Mandemakers J.J., Snieder H., Stolk R.P., Smidt N. (2015). Representativeness of the LifeLines Cohort Study. PLoS ONE.

[26] Nederland S.V. (1998). Zo Eet Nederland: Resultaten Van De Voedselconsumptiepeiling 1997–1998.

[27] Iwani N.A., Jalaludin M.Y., Zin R.M., Fuziah M.Z., Hong J.Y., Abqariyah Y., Mokhtar A.H., Wan Nazaimoon W.M. (2017). Triglyceride to HDL-C Ratio is Associated with Insulin Resistance in Overweight and Obese Children. Sci. Rep..

[28] Hoving E.B., Jansen G., Volmer M., Van Doormaal J.J., Muskiet F.A. (1988). Profiling of Plasma Cholesterol Ester and Triglyceride Fatty Acids as their Methyl Esters by Capillary Gas Chromatography, Preceded by a Rapid Aminopropyl-Silica Column Chromatographic Separation of Lipid Classes. J. Chromatogr..

[29] Folch J., Lees M., Sloane Stanley G.H. (1957). A Simple Method for the Isolation and Purification of Total Lipides from Animal Tissues. J. Biol. Chem..

[30] Kaluzny M.A., Duncan L.A., Merritt M.V., Epps D.E. (1985). Rapid Separation of Lipid Classes in High Yield and Purity using Bonded Phase Columns. J. Lipid Res..

[31] Robberecht E., Koletzko B., Christophe A. (1997). Several Mechanisms Contribute to the Abnormal Fatty Acid Composition of Serum Phospholipids and Cholesterol Esters in Cholestatic Children with Extrahepatic Biliary Atresia. Prostaglandins Leukot. Essent. Fatty Acids.

[32] Smedman A.E., Gustafsson I.B., Berglund L.G., Vessby B.O. (1999). Pentadecanoic Acid in Serum as a Marker for Intake of Milk Fat: Relations between Intake of Milk Fat and Metabolic Risk Factors. Am. J. Clin. Nutr..

[33] Wolk A., Vessby B., Ljung H., Barrefors P. (1998). Evaluation of a Biological Marker of Dairy Fat Intake. Am. J. Clin. Nutr..

[34] Wolk A., Furuheim M., Vessby B. (2001). Fatty Acid Composition of Adipose Tissue and Serum Lipids are Valid Biological Markers of Dairy Fat Intake in Men. J. Nutr..

[35] Golley R.K., Hendrie G.A. (2014). Evaluation of the Relative Concentration of Serum Fatty Acids C14:0, C15:0 and C17:0 as Markers of Children’s Dairy Fat Intake. Ann. Nutr. Metab..

[36] Yakoob M.Y., Shi P., Hu F.B., Campos H., Rexrode K.M., Orav E.J., Willett W.C., Mozaffarian D. (2014). Circulating Biomarkers of Dairy Fat and Risk of Incident Stroke in U.S. Men and Women in 2 Large Prospective Cohorts. Am. J. Clin. Nutr..

[37] Smit L.A., Baylin A., Campos H. (2010). Conjugated Linoleic Acid in Adipose Tissue and Risk of Myocardial Infarction. Am. J. Clin. Nutr..

[38] Jiang J., Wolk A., Vessby B. (1999). Relation between the Intake of Milk Fat and the Occurrence of Conjugated Linoleic Acid in Human Adipose Tissue. Am. J. Clin. Nutr..

[39] Rosell M., Johansson G., Berglund L., Vessby B., de Faire U., Hellenius M.L. (2004). Associations between the Intake of Dairy Fat and Calcium and Abdominal Obesity. Int. J. Obes. Relat. Metab. Disord..

[40] Brouwer-Brolsma E., Sluik D., Singh-Povel C., Feskens E. (2018). Dairy shows Different Associations with Abdominal and BMI-Defined Overweight: Cross-Sectional Analyses Exploring a Variety of Dairy Products. Nutr. Metab. Cardiovasc. Dis..

[41] Kratz M., Baars T., Guyenet S. (2013). The Relationship between High-Fat Dairy Consumption and Obesity, Cardiovascular, and Metabolic Disease. Eur. J. Nutr..

[42] Mozaffarian D., de Oliveira Otto M.C., Lemaitre R.N., Fretts A.M., Hotamisligil G., Tsai M.Y., Siscovick D.S., Nettleton J.A. (2013). Trans-Palmitoleic Acid, Other Dairy Fat Biomarkers, and Incident Diabetes: The Multi-Ethnic Study of Atherosclerosis (MESA). Am. J. Clin. Nutr..

[43] Hansen C.P., Berentzen T.L., Halkjaer J., Tjonneland A., Sorensen T.I., Overvad K., Jakobsen M.U. (2012). Intake of Ruminant Trans Fatty Acids and Changes in Body Weight and Waist Circumference. Eur. J. Clin. Nutr..

[44] Kratz M., Marcovina S., Nelson J.E., Yeh M.M., Kowdley K.V., Callahan H.S., Song X., Di C., Utzschneider K.M. (2014). Dairy Fat Intake is Associated with Glucose Tolerance, Hepatic and Systemic Insulin Sensitivity, and Liver Fat but Not Beta-Cell Function in Humans. Am. J. Clin. Nutr..

[45] Iggman D., Arnlov J., Vessby B., Cederholm T., Sjogren P., Riserus U. (2010). Adipose Tissue Fatty Acids and Insulin Sensitivity in Elderly Men. Diabetologia.

[46] Brouwer-Brolsma E.M., Sluik D., Singh-Povel C.M., Feskens E.J.M. (2018). Dairy Product Consumption is Associated with Pre-Diabetes and Newly Diagnosed Type 2 Diabetes in the Lifelines Cohort Study. Br. J. Nutr..

[47] Wang H., Steffen L.M., Vessby B., Basu S., Steinberger J., Moran A., Jacobs D.R., Hong C.P., Sinaiko A.R. (2011). Obesity Modifies the Relations between Serum Markers of Dairy Fats and Inflammation and Oxidative Stress among Adolescents. Obesity (Silver Spring).

[48] Mozaffarian D., Cao H., King I.B., Lemaitre R.N., Song X., Siscovick D.S., Hotamisligil G.S. (2010). Trans-Palmitoleic Acid, Metabolic Risk Factors, and New-Onset Diabetes in U.S. Adults: A Cohort Study. Ann. Intern. Med..

[49] Mathieu P., Lemieux I., Després J. (2010). Obesity, Inflammation, and Cardiovascular Risk. Clin. Pharmacol. Ther..

[50] Ridker P.M. (2004). High-Sensitivity C-Reactive Protein, Inflammation, and Cardiovascular Risk: From Concept to Clinical Practice to Clinical Benefit. Am. Heart J..

[51] Li Y., Zhong X., Cheng G., Zhao C., Zhang L., Hong Y., Wan Q., He R., Wang Z. (2017). Hs-CRP and all-Cause, Cardiovascular, and Cancer Mortality Risk: A Meta-Analysis. Atherosclerosis.

[52] Choi H.K., Liu S., Curhan G. (2005). Intake of Purine-Rich Foods, Protein, and Dairy Products and Relationship to Serum Levels of Uric Acid: The Third National Health and Nutrition Examination Survey. Arthritis Rheum..

[53] Chen J.H., Chuang S.Y., Chen H.J., Yeh W.T., Pan W.H. (2009). Serum Uric Acid Level as an Independent Risk Factor for all-Cause, Cardiovascular, and Ischemic Stroke Mortality: A Chinese Cohort Study. Arthritis Rheum..

[54] Niskanen L.K., Laaksonen D.E., Nyyssonen K., Alfthan G., Lakka H.M., Lakka T.A., Salonen J.T. (2004). Uric Acid Level as a Risk Factor for Cardiovascular and all-Cause Mortality in Middle-Aged Men: A Prospective Cohort Study. Arch. Intern. Med..

[55] Ruggiero C., Cherubini A., Ble A., Bos A.J., Maggio M., Dixit V.D., Lauretani F., Bandinelli S., Senin U., Ferrucci L. (2006). Uric Acid and Inflammatory Markers. Eur. Heart J..

[56] Frohlich M., Imhof A., Berg G., Hutchinson W.L., Pepys M.B., Boeing H., Muche R., Brenner H., Koenig W. (2000). Association between C-Reactive Protein and Features of the Metabolic Syndrome: A Population-Based Study. Diabetes Care.

[57] Hodge A.M., English D.R., O’Dea K., Sinclair A.J., Makrides M., Gibson R.A., Giles G.G. (2007). Plasma Phospholipid and Dietary Fatty Acids as Predictors of Type 2 Diabetes: Interpreting the Role of Linoleic Acid. Am. J. Clin. Nutr..

[58] Sluik D., Geelen A., de Vries J.H., Eussen S.J., Brants H.A., Meijboom S., van Dongen M.C., Bueno-de-Mesquita H.B., Wijckmans-Duysens N.E., van’t Veer P. (2016). A National FFQ for the Netherlands (the FFQ-NL 1.0): Validation of a Comprehensive FFQ for Adults. Br. J. Nutr..

[59] Brouwer-Brolsma E.M., Streppel M.T., van Lee L., Geelen A., Sluik D., van de Wiel A.M., de Vries J.H.M., van’t Veer P., Feskens E.J.M. (2017). A National Dietary Assessment Reference Database (NDARD) for the Dutch Population: Rationale Behind the Design. Nutrients.

[60] Glaser C., Demmelmair H., Koletzko B. (2010). High-Throughput Analysis of Fatty Acid Composition of Plasma Glycerophospholipids. J. Lipid Res..

