# Supplementary material for: Potential Biomarkers for Fat from Dairy and Fish and Their Association with Cardiovascular Risk Factors: Cross-sectional Data from the LifeLines Biobank and Cohort Study
Source: Nutrients. 2019 May 17;11(5):1099. doi: 10.3390/nu11051099 (PMC6566248; doi:10.3390/nu11051099)
Supplement: Supplementary file 1 [file nutrients-11-01099-s001.pdf]

Supplementary data

Table S1: Cross-sectional association between circulating fatty acids and dairy fat intake

Table S2: Cross-sectional association between circulating fatty acids and fish fat intake

Table S3: Association between the circulating fatty acids EPA and DHA and fish fat intake

Table S4: Association between fatty acid markers from plasma triglycerides (TG) and cardiovascular risk factors.

Table S5: Association between fatty acid markers from plasma phospholipids (PL) and cardiovascular risk factors.

Table S6: Association between dairy fat intake and cardiovascular risk factors

Table S7: Association between fish fat intake and cardiovascular risk factors

Table S8A-C: Papers investigating the association between dairy fat biomarkers and cardiovascular health (based on an illustrative search)

References

Table S1: Cross-sectional association between circulating fatty acids and dairy fat intake

Total dairy Fat (g/d)	Fatty acids											
	C14:0		C15:0		C17:0		Trans-C16:1(n-7)		Trans-C18:1(n-7)		CLA	
	**β**	**p-value**	**β**	**p-value**	**β**	**p-value**	**β**	**p-value**	**β**	**p-value**	**β**	**p-value**
Plasma TG												
Model 1	0.222	<0.001	0.257	<0.001	0.030	0.43	0.188	<0.001	0.261	<0.001	0.171	<0.001
Model 2	0.175	<0.001	0.250	<0.001	0.048	0.15	0.205	<0.001	0.248	<0.001	0.188	<0.001
Model 3	0.162	<0.001	0.231	<0.001	0.043	0.18	0.189	<0.001	0.229	<0.001	0.173	<0.001
Model 4	0.167	<0.001	0.230	<0.001	0.040	0.21	0.194	<0.001	0.230	<0.001	0.175	<0.001
Model 5	0.183	<0.001	0.240	<0.001	0.040	0.20	0.191	<0.001	0.218	<0.001	0.182	<0.001
Model 6	0.168	<0.001	0.230	<0.001	0.041	0.20	0.195	<0.001	0.229	<0.001	0.175	<0.001
Model 7	0.204	<0.001	0.231	<0.001	0.042	0.27	0.193	<0.001	0.236	<0.001	0.182	<0.001
Plasma PL												
Model 1	0.198	<0.001	0.192	<0.001	0.127	<0.001	0.085	0.02	0.232	<0.001	0.228	<0.001
Model 2	0.202	<0.001	0.189	<0.001	0.119	<0.001	0.118	<0.001	0.239	<0.001	0.242	<0.001
Model 3	0.170	<0.001	0.162	<0.001	0.092	0.003	0.105	0.001	0.219	<0.001	0.217	<0.001
Model 4	0.174	<0.001	0.164	<0.001	0.090	0.004	0.110	<0.001	0.214	<0.001	0.220	<0.001
Model 5	0.182	<0.001	0.165	<0.001	0.127	<0.001	0.101	0.001	0.197	<0.001	0.211	<0.001
Model 6	0.174	<0.001	0.164	<0.001	0.090	0.003	0.111	<0.001	0.214	<0.001	0.220	<0.001
Model 7	0.177	<0.001	0.166	<0.001	0.094	0.003	0.109	<0.001	0.214	<0.001	0.229	<0.001

Model 1: Crude model; **Model 2**: Adjusted for energy intake; **Model 3:** Adjusted for Model 2 + age, sex, education and smoking; **Model 4:** Adjusted for model 2 + age, sex and BMI; **Model 5**: Adjusted for model 4 + total carbohydrates and Fat; **Model 6:** Adjusted for Model 4 + fish fat intake; **Model 7:** Adjusted for model 4 + Total Triglycerides + Total Cholesterol

Dependent: Dairy fat intake, independent: Circulating fatty acids. Associations between fatty acid status and dairy fat intake are reported as standardized β's.
Abbreviations: C14:0, Myristic acid; C15:0, Pentadecanoic acid; C17:0, Heptadecanoic acid; Trans-C16:1(n-7), Trans-Palmitoleic acid; Trans-C18:1(n-7), Vaccenic acid; CLA, Conjugated Linoleic acid; TG, Triglycerides; PL, Phospholipids; BMI, Body Mass Index

Table S2: Cross-sectional association between circulating fatty acids and fish fat intake

Total fish Fat (g/d)	Fatty acids											
	C14:0		C15:0		C17:0		Trans-C16:1(n-7)		Trans-C18:1(n-7)		CLA	
	β	p-value	β	p-value	β	p-value	β	p-value	β	p-value	β	p-value
Plasma TG												
Model 1	-0.094	0.01	-0.045	0.22	0.080	0.04	0.022	0.60	-0.018	0.61	-0.093	0.01
Model 2	-0.101	0.01	-0.045	0.21	0.082	0.03	0.024	0.57	-0.020	0.58	-0.091	0.01
Model 3	-0.118	0.001	-0.069	0.06	0.093	0.02	0.001	0.98	-0.045	0.23	-0.106	0.004
Model 4	-0.104	0.01	-0.062	0.09	0.075	0.05	0.017	0.69	-0.030	0.42	-0.097	0.01
Model 5	-0.095	0.01	-0.045	0.21	0.087	0.02	0.019	0.63	-0.032	0.38	-0.101	0.004
Model 6	-0.104	0.01	-0.062	0.10	0.076	0.05	0.022	0.61	-0.027	0.48	-0.098	0.01
Model 7	-0.068	0.09	-0.058	0.11	0.023	0.61	0.008	0.85	-0.019	0.61	-0.082	0.02
Plasma PL												
Model 1	-0.064	0.07	-0.008	0.83	0.067	0.07	0.034	0.35	-0.065	0.89	-0.070	0.05
Model 2	-0.064	0.08	-0.008	0.83	0.066	0.07	0.039	0.29	-0.004	0.91	-0.068	0.06
Model 3	-0.087	0.02	-0.016	0.65	0.063	0.09	0.026	0.49	-0.024	0.53	-0.089	0.02
Model 4	-0.076	0.04	-0.020	0.59	0.049	0.19	0.034	0.36	-0.020	0.60	-0.078	0.03
Model 5	-0.068	0.06	-0.007	0.84	0.098	0.01	0.041	0.26	-0.014	0.72	-0.097	0.01
Model 6	-0.076	0.04	-0.017	0.65	0.051	0.17	0.037	0.33	-0.016	0.67	-0.079	0.04
Model 7	-0.077	0.04	-0.036	0.33	0.037	0.34	0.020	0.59	-0.029	0.44	-0.070	0.06

Model 1: Crude model; **Model 2**: Adjusted for energy intake; **Model 3:** Adjusted for Model 2 + age, sex, education and smoking; **Model 4:** Adjusted for Model 2 + age, sex and BMI; **Model 5**: Adjusted for model 4 + total carbohydrates and Fat; **Model 6:** Adjusted for Model 4 + dairy fat intake; **Model 7:** Adjusted for model 4 + Total Triglycerides + Total Cholesterol

Dependent: fish fat intake, independent: fatty acids. Associations between fatty acid status and fish fat intake are reported as standardized β's. Abbreviations: C14:0, Myristic acid; C15:0, Pentadecanoic acid; C17:0, Heptadecanoic acid; Trans-C16:1(n-7), Trans-Palmitoleic acid; Trans-C18:1(n-7), Vaccenic acid; CLA, Conjugated Linoleic acid; TG, Triglycerides; PL, Phospholipids; BMI, Body Mass Index

Table S3: Association between the circulating fatty acids EPA and DHA and fish fat intake

Total fish Fat (g/d)	Omega-3 fatty acids			
	EPA		DHA	
	**β**	**p-value**	**β**	**p-value**
Plasma TG				
Model 1	0.277	<0.001	0.425	<0.001
Model 2	0.283	<0.001	0.440	<0.001
Model 3	0.272	<0.001	0.445	<0.001
Plasma PL				
Model 1	0.256	<0.001	0.457	<0.001
Model 2	0.258	<0.001	0.465	<0.001
Model 3	0.248	<0.001	0.464	<0.001

Model 1: Crude model; **Model 2:** Adjusted for model 1 + energy intake; **Model 3**: Adjusted for Model 2 + age and sex, education and smoking

Dependent: fish fat intake, independent: fatty acids. Associations between fatty acid status and fish fat intake are reported as standardized β's.
Abbreviations: DHA, docosahexaenoic acid; EPA: eicosapentaenoic acid; PL, Phospholipids; TG, Triglycerides

Table S4: Association between fatty acid markers from plasma triglycerides (TG) and cardiovascular risk factors

	**C14:0**		**C15:0**		**C17:0**		**Trans-C16:1(n-7)**		**Trans-C18:1(n-7)**		**CLA**	
	**Model S1**	**Model S2**	**Model S1**	**Model S2**	**Model S1**	**Model S2**	**Model S1**	**Model S2**	**Model S1**	**Model S2**	**Model S1**	**Model S2**
**Dependent variable**												
Weight (kg)	**0.074***	-0.025	**-0.079***	-0.079	**-0.116****	0.022	-0.032	-0.027	0.011	-0.026	0.000	-0.040
Waist circumference	**0.094****	-0.041	**-0.096****	**-0.107*****	**-0.144*****	0.039	-0.035	-0.040	0.023	-0.033	-0.007	**-0.064***
BMI (kg/m^2)	**0.081***	-0.066	**-0.121****	**-0.126*****	**-0.173*****	0.022	0.078	-0.052	-0.010	**-0.067***	-0.015	**-0.075***
SBP (mmHg)	0.032	-0.047	-0.059	**-0.066***	**-0.074***	0.039	-0.024	-0.036	-0.013	-0.050	-0.044	-0.080
DBP (mmHg)	**0.073***	0.001	**-0.064***	**-0.067***	**-0.088***	0.005	-0.008	-0.003	0.014	-0.013	0.015	-0.013
Total C (mmol/L)	**0.145*****	0.009	-0.025	-0.017	**-0.311*****	**-0.147*****	0.028	0.063	**0.104****	0.070*	0.057	0.015
HDL-c(mmol/L)	**-0.222*****	-0.057	**-0.072***	**-0.060***	**0.156*****	**-0.113****	-0.007	-0.010	-0.063	0.005	**-0.093****	-0.023
LDL-c (mmol/L)	**0.150*****	0.039	-0.001	0.005	**-0.273*****	**-0.133****	0.016	0.047	**0.093***	0.062	0.056	0.019
Total TG	**0.384*****	-	0.004	-	**-0.543*****	-	-0.042	-	**0.126*****	-	**0.141*****	-
TG/HDL-c ratio	**0.368*****	0.016	0.030	**0.022***	**-0.462*****	**0.049****	-0.027	0.006	**0.121*****	0.000	**0.140*****	0.007
Glucose (mmol/L)	**0.106****	0.014	-0.044	-0.049	**-0.100****	0.038	-0.027	-0.016	0.035	0.000	0.017	-0.025
HbA1c (%)	**0.093****	0.034	-0.020	-0.039	-0.023	0.080	-0.013	-0.021	0.011	-0.022	-0.023	-0.060
Hs-CRP (mg/L)[1]	0.053	-0.038	**-0.103***	**-0.110***	**-0.188*****	**-0.119***	-0.060	-0.070	-0.047	-0.085	-0.018	-0.065
Creatinine (umol/L)	0.015	-0.038	-0.039	-0.023	**-0.067***	0.022	0.018	0.028	0.001	-0.008	-0.001	-0.012
Uric acid (mmol/L)[1]	0.084	**-0.134***	**-0.150****	**-0.163*****	**-0.230*****	0.052	-0.090	-0.085	-0.091	**-0.140****	-0.027	-0.079
Ureum (mmol/L)[1]	0.016	0.001	**0.130****	**0.121***	0.087	**0.138***	0.109	0.097	**0.143****	**0.137***	0.082	0.079

Model S1: Adjusted for age, sex, education and smoking**; Model S2:** Adjusted for age, sex and triglycerides.

Dependent: cardiovascular risk factors, independent: fatty acid status. Associations between fatty acid status and cardiovascular risk factor are reported as standardized β's. P-value: * = ≤ 0.05, ** = < 0.01, *** = < 0.001. Abbreviations: BMI, body mass index; SBP, systolic blood pressure; DBP, diastolic blood pressure; Total C; total cholesterol; HDL-c, high density lipoprotein cholesterol; LDL-c, Low density lipoprotein cholesterol; Total TG, total triglycerides; TG/HDL-c ratio, total triglycerides/HDL cholesterol; HbA1c, Hemoglobin A1C; Hs-CRP, High sensitive C-reactive protein. [1.] Hs-Crp data was available in n = 412, Uric acid and ureum data were available in n = 348

Table S5: Association between fatty acid markers from plasma phospholipids (PL) and cardiovascular risk factors.

	C14:0		C15:0		C17:0		Trans-C16:1(n-7)		Trans-C18:1(n-7)		CLA	
	Model S1	Model S2	Model S1	Model S2	Model S1	Model S2	Model S1	Model S2	Model S1	Model S2	Model S1	Model S2
Dependent variable												
Weight (kg)	-0.002	-0.015	-0.056	-0.019	-0.150***	-0.102**	-0.011	0.012	-0.056	-0.047	0.012	-0.024
Waist circumference	0.001	-0.023	-0.090**	-0.047	-0.176***	-0.120***	-0.052	-0.027	-0.070*	-0.063*	-0.008	-0.058
BMI (kg/m^2)	-0.018	-0.040	-0.087	-0.017	-0.186***	-0.120**	-0.049	-0.021	-0.099*	-0.087*	-0.025	-0.079*
SBP (mmHg)	-0.026	-0.046	-0.043	-0.017	-0.074*	-0.026	-0.049	-0.041	-0.087*	-0.083*	-0.048	-0.088*
DBP (mmHg)	-0.005	-0.030	-0.067*	-0.038	-0.128***	-0.078*	-0.039	-0.021	-0.063	-0.057	0.012	-0.023
Total C (mmol/L)	0.117**	-	-0.087*	-	-0.253***	-	-0.052	-	0.006	-	0.136***	-
HDL-c(mmol/L)	-0.012	-0.022	0.030	-0.022	-0.063	-0.109***	-0.015	-0.057*	0.022	-0.015	0.018	0.043
LDL-c (mmol/L)	0.120**	0.010	-0.073*	0.005	-0.184***	0.051***	-0.025	0.019	0.011	-0.001	0.100**	-0.028*
Total TG	0.053	-	-0.146***	-	-0.232***	-	-0.114**	-	-0.073*	-	0.120**	-
TG/HDL-c ratio	0.042	0.007	-0.119***	0.010	-0.147***	0.043***	-0.076*	0.025*	-0.061	0.008	0.080*	-0.018
Glucose (mmol/L)	0.029	0.026	-0.060	-0.031	-0.107**	-0.085*	-0.023	0.005	-0.084*	-0.069*	-0.044	-0.065*
HbA1c (%)	-0.019	-0.024	-0.030	-0.019	-0.029	-0.031	-0.018	-0.009	-0.084*	-0.081**	-0.023	-0.037
Hs-CRP (mg/L)[1]	-0.037	-0.056	-0.081	-0.051	-0.222***	-0.200***	-0.110*	-0.102*	-0.172***	-0.168*	0.015	-0.028
Creatinine (umol/L)	-0.041	-0.044	-0.005	0.020	-0.109***	-0.060*	0.002	0.022	-0.055	-0.032	-0.006	-0.022
Uric acid (mmol/L)[1]	-0.087	-0.106*	-0.119*	-0.076	-0.265***	-0.197***	-0.108*	-0.039	-0.197***	-0.128**	-0.047	-0.073
Ureum (mmol/L)[1]	0.012	0.020	0.188**	0.188***	0.091	0.088	0.096	0.104	0.093	0.105	0.028	0.037

Model S1: Adjusted for age, sex, education and smoking**; Model S2:** Adjusted for age, sex, triglycerides and cholesterol.

Dependent: cardiovascular risk factors, independent: fatty acid status. Associations between fatty acid status and cardiovascular risk factor are reported as standardized β's. P-value: * = ≤ 0.05, ** = < 0.01, *** = < 0.001. Abbreviations: BMI, body mass index; SBP, systolic blood pressure; DBP, diastolic blood pressure; Total C; total cholesterol; HDL-c, high density lipoprotein cholesterol; LDL-c, Low density lipoprotein cholesterol; Total TG, total triglycerides; TG/HDL-c ratio, total triglycerides/HDL cholesterol; HbA1c, Hemoglobin A1C; Hs-CRP, High sensitive C-reactive protein. [1.] Hs-Crp data was available in n = 412, Uric acid and ureum data were available in n = 348

Table S6: Association between dairy fat intake and cardiovascular risk factors

	Dairy fat intake (g/d)					
	**Model 1**	**Model 2**	**Model 3**	**Model 4**	**Model 5**	**Model 6**
Age (years)	**0.355*** **					
Sex	**0.083***					
Weight (kg)	-0.003	0.022	0.043	0.037	0.043	0.042
Waist circumference	**0.094***	0.007	0.023	0.014	0.023	0.025
BMI (kg/m^2)	0.048	-0.022				
SBP (mmHg)	**0.103***	-0.043	-0.048	-0.044	-0.048	-0.046
DBP (mmHg)	**0.108****	0.021	0.023	0.012	0.024	0.024
Total C (mmol/L)	**0.118****	-0.003	0.006	0.000	0.006	
HDL-c(mmol/L)	**0.112****	0.051	0.051	-0.004	0.053	0.036
LDL-c (mmol/L)	**0.086***	-0.015	-0.003	0.012	-0.003	-0.011
Total TG	0.009	-0.024	-0.026	-0.009	-0.028	
TG/HDL-c ratio	-0.037	-0.038	-0.039	-0.006	-0.041	-0.014
Glucose (mmol/L)	**0.085***	-0.036	-0.024	-0.055	-0.024	-0.018
HbA1c (%)	**0.215*** **	0.020	0.027	0.030	0.027	0.031
Hs-CRP (mg/L)[1]	0.001	0.005	-0.008	0.004	-0.007	-0.012
Creatinine (umol/L)	-0.030	-0.020	-0.026	-0.030	-0.027	-0.023
Uric acid (mmol/L)[1]	**-0.172****	**-0.122***	**-0.107***	**-0.106***	-0.101	-0.058
Ureum (mmol/L)[1]	**0.188****	**0.183****	**0.186****	**0.184****	**0.186****	**0.184****

Model 1: Adjusted for energy intake; **Model 2**: Model 1 + age, sex, education and smoking; **Model 3**: Model 1 + age, sex and BMI; **Model 4**: Model 3 + total carbohydrates and total fat; **Model 5**: Model 3 + total fish fat intake; **Model 6**: Model 3 + total triglycerides and total cholesterol.

Dependent: cardiovascular risk factors, independent: dairy fat intake. Associations between dairy fat intake and cardiovascular risk factor are reported as standardized β's. P-value: * = ≤ 0.05, ** = < 0.01, *** = < 0.001. Abbreviations: BMI, body mass index; SBP, systolic blood pressure; DBP, diastolic blood pressure; Total C; total cholesterol; HDL-c, high density lipoprotein cholesterol; LDL-c, Low density lipoprotein cholesterol; Total TG, total triglycerides; TG/HDL-c ratio, total triglycerides/HDL cholesterol; HbA1c, Hemoglobin A1C; Hs-CRP, High sensitive C-reactive protein. [1.] Hs-Crp data was available in n = 412, Uric acid and ureum data were available in n = 348

Table S7: Association between fish fat intake and cardiovascular risk factors

	Fish fat intake (g/d)					
	**Model 1**	**Model 2**	**Model 3**	**Model 4**	**Model 5**	**Model 6**
Age (years)	**0.077***					
Sex	0.004					
Weight (kg)	-0.041	-0.036	0.007	0.003	0.007	0.003
Waist circumference	-0.022	-0.035	0.000	-0.001	0.001	0.007
BMI (kg/m^2)	-0.045	-0.048				
SBP (mmHg)	0.032	0.014	0.013	0.013	0.012	0.022
DBP (mmHg)	0.041	0.020	0.029	0.012	0.029	0.038
Total C (mmol/L)	0.019	-0.015	0.001	-0.011	0.002	
HDL-c(mmol/L)	**0.100****	**0.080***	**0.075***	0.042	**0.076***	0.018
LDL-c (mmol/L)	0.018	0.043	0.007	0.009	0.007	-0.001
Total TG	**-0.117****	**-0.120****	**-0.109****	**-0.110****	**-0.109****	
TG/HDL-c ratio	**-0.126*****	**-0.121*****	**-0.110*****	**-0.098****	**-0.111*****	-0.006
Glucose (mmol/L)	0.000	-0.030	-0.006	-0.014	-0.006	0.016
HbA1c (%)	0.016	-0.026	-0.013	-0.004	-0.012	0.001
Hs-CRP (mg/L)[1]	-0.085	-0.079	-0.047	-0.047	-0.047	-0.033
Creatinine (umol/L)	-0.013	-0.012	-0.015	-0.021	-0.016	-0.002
Uric acid (mmol/L)[1]	0.097	**0.102***	0.081	0.079	0.076	**0.102****
Ureum (mmol/L)[1]	-0.030	-0.019	-0.012	-0.015	-0.001	-0.011

Model 1: Adjusted for energy intake; **Model 2**: Model 1 + age, sex, education and smoking; **Model 3**: Model 1 + age, sex and BMI; **Model 4**: Model 3 + total carbohydrates and total fat; **Model 5**: Model 3 + total dairy fat intake; **Model 6**: Model 3 + total triglycerides and total cholesterol.

Dependent: cardiovascular risk factors, independent: fish fat intake. Associations between dairy fat intake and cardiovascular risk factor are reported as standardized β's. P-value: * = ≤ 0.05, ** = < 0.01, *** = < 0.001. Abbreviations: BMI, body mass index; SBP, systolic blood pressure; DBP, diastolic blood pressure; Total C; total cholesterol; HDL-c, high density lipoprotein cholesterol; LDL-c, Low density lipoprotein cholesterol; Total TG, total triglycerides; TG/HDL-c ratio, total triglycerides/HDL cholesterol; HbA1c, Hemoglobin A1C; Hs-CRP, High sensitive C-reactive protein. [1.] Hs-Crp data was available in n = 412, Uric acid and ureum data were available in n = 348

Table S8A: Papers investigating the association between dairy fat biomarkers and cardiovascular risk factors (based on an illustrative search)

								Biomarkers[1,2]				
**Fraction**	**Plasma/Serum**	**Paper**	**Population**	**n**	**Outcome**		**+/-[3]**	**C14:0**	**C15:0**	**C15:0+ C17:0**	**C17:0**	**Trans-C16:1(n-7)**
CE+TG+PL+FFA	Plasma	-	-	-	-							
	Serum	Santaren et al. (2014)[1]	Hispanic, African American and Non-Hispanic white adults 40-60 yr	659	Insulin resistance by intravenous glucose-tolerance test (insulin sensitivity index)	r	+		0.14***			-0.12**
						β	+		0.84*			-0.42
					β-cell dysfunction by intravenous glucose-tolerance test (product of the insulin sensitivity index and acute insulin response)	r	+		0.11**			-
						β	+		2.21*			0.10
CE	Plasma	-	-	-	-	-		-	-	-	-	-
	Serum	Smedman et al. (1999)[2]	70-year old men	66	Weight	r	+		- 0.36**			
					BMI	r	+		- 0.39***			
					WC	r	+		- 0.28*			
					HC	r	+		- 0.32**			
					HDL triacylglycerol	r	+		- 0.23			
					Insulin sensitivity by clamp (insulin sensitivity index (glucose disposal/actual insulin concentration) *100)	r	+		0.21			
TG	Plasma/Serum	-	-	-	-	-		-	-	-	-	-
PL	Plasma	Mozaffarian et al. (2010)[3]	Cardio vascular Health study, adults from 4 US communities	3736	BMI	Δ%[a,c]	+					-1.8%*
					WC	Δ%	+					-1.8%**
					LDL	Δ%	0					+2.4%
					HDL	Δ%	+					+1.9%*
					Total Triglycerides	Δ%	+					-19.0%***
					Total:HDL ratio	Δ%	+					-4.7%***
					CRP	Δ%	+					-13.8%*
					Fasting glucose	Δ%	0					-0.96%
					Fasting insulin	Δ%	+					-13.3%***
					Insulin resistance by HOMA-IR	Δ%	+					-16.7%**
		Mozaffarian et al. (2013)[4]	US adults (45-84 yr) from the Multi-ethnic study of	2617	SBP	Δ%[a,c]	+					-2.4%*
					DBP	Δ%	+					-2.9%***
					LDL	Δ%	-					+6.4%***
					HDL	Δ%	0					-1.19
					Total Triglycerides	Δ%	+					-19.1%***

			athero-sclerosis		Total:HDL ratio	Δ%	0					+1.5%
					Fasting glucose	Δ%	0					-1.2%
					Fasting insulin	Δ%	+					-9.1%***
		Nestel et al. (2014)[5]	Overweight and obese subjects with metabolic syndrome	86	insulin sensitivity index by OGTT (Matsuda index)	β	+				0.30*	0.02
					Insulin resistance (Fasting Insulin) by OGTT	β	+				-2.41*	-0.76
					Insulin resistance (HOMA-IR) by OGTT	β	+				-0.70*	-0.17
		Oliveira et al. (2013)[6]	US adults (45-84 yr) from the Multi-ethnic study of athero-sclerosis	2837	SBP	Δ%[a,c]	+	+2.5%*	-4.0%***			-1.18%*
					DBP	Δ%	+	+1.4%*	-4.1%***			-2.1%**
					LDL	Δ%	0	+1.9%	-1.11%			+4.4*
					HDL	Δ%	0	-0.2%	+0.4%			-0.6%
					Total: HDL ratio	Δ%	0	+5.0%**	-3.4%			+1.0%
					Total Triglycerides	Δ%	+	+29.5%***	-15.0%***			-17.7%***
					CRP	Δ%	0	0%	0%			+2.8%
	Serum	Kratz et al. (2014)[7]	Population without fatty liver disease	32	Fasting glucose	β	+		-1.84*		-0.80*	-1.31*
					AUC glucose by OGTT	β	+		-34.27*		-23.94*	-36.02*
					Incremental AUC glucose by OGTT	β	+		-9.9		-13.26*	-18.68
					Liver fat (liver-spleen ratio)	β	+		2.39		2.13*	3.56*
					Hepatic insulin resistance (basal hepatic glucose * fasting plasma insulin)	β	+		-1.21		-2.68	-5.95*
					Systemic insulin resistance (LL)	β	0		-0.60		5.93	11.2*
					Systemic insulin resistance (HL)	β	0		-0.78		5.72	10.97*
					Pancreatic β-cell function (AIRg)	β	0		2.12		4.35	-1.67
					Pancreatic β-cell function (oral DI)	β	0		2.73		3.82	4.34
					Pancreatic β-cell function (IV DI)	β	0		1.68		6.24	1.24
		Rosell et al. (2004)[8]	Healthy men (63-year old)	299	Abdominal obesity	r	+	0.09	-0.22***		-0.30***	
		Smedman et al. (1999)[2]	Men (70-years old)	66	Weight	r	0		-0.21			
					HC	r	+		-0.25*			
					Apo-B	r	0		-0.27			
		Warensjö et al. (2010)[9]	Swedish general population	1000	SBP	r	+		-0.05	-0.10***	-0.11***	
					DBP	r	+		-0.06	-0.13**	-0.16**	
					BMI	r	+		-0.08*	-0.14**	-0.14**	
					Total cholesterol	r	+		-0.10***	-0.04	0.008	

					Total Triglycerides	r	+		-0.18**	-0.23**	-0.22*	
					Fasting glucose	r	+		-0.05	-0.13**	-0.16*	
FFA	Plasma/Serum	Kratz et al. (2014)[7]	Population without fatty liver disease	32	Fasting glucose	β	+		-1.09*		-0.54	-1.23
					AUC glucose by OGTT	β	+		-32.43*		-18.07*	-35.25
					Incremental AUC glucose by OGTT	β	+		-18.00*		-10.96*	-18.95
					Liver fat (liver-spleen ratio)	β	+		2.55*		1.80*	3.20
					Hepatic insulin resistance (basal hepatic glucose * fasting plasma insulin)	β	0		-2.21		-0.74	-4.79
					Systemic insulin resistance (LL)	β	0		7.24		1.15	7.95
					Systemic insulin resistance (HL)	β	0		6.89		1.10	8.14
					Pancreatic β-cell function (AIRg)	β	0		-2.32		2.29	-3.49
					Pancreatic β-cell function (oral DI)	β	0		3.75		1.30	-1.82
					Pancreatic β-cell function (IV DI)	β	0		2.15		4.05	4.82
Erythrocytes	-	-	-	-	-	-		-	-	-	-	-
AT	-	Castro-Webb et al. (2012)[10]	Costa-Rican adults	1744 (232 cases)	Type 2 diabetes	PR[b]	0			1.33 (0.88-2.11)		1.05 (0.66-1.66)
		Iggman et al. (2010)[11]	Elderly men	795	Insulin sensitivity by clamp (glucose infusion rate)	r	+	0.32***	0.07		0.21***	
						β	+	0.35**	0.04		3.5*	
					Insulin sensitivity (HOMA-IR)	r	+	-0.27***	-0.04		-0.21***	
						β	+	-0.10*	0.10		-1.4***	
		Rosell et al. (2004)[12]	Healthy men (63-year old)	297	Abdominal obesity	r	+	-0.30***	-0.20***		-0.31***	
		Smit et al. (2010)[13]	Costa-Rican Adults	1785	BMI	β	-					0.37***
					WC	β	-					0.95***
					SS	β	+					-2.27***
					TS	β	+					-1.38***
					SUS	β	+					-1.67***

Significance level: *= p <0.05, **= p <0.01, ***= p <0.001.

[a]. Relationships of the dairy fatty acid with cardiovascular risk factors were evaluated using linear regression; [b.]Highest versus lowest quintile; [c]. Δ% is calculated by the authors of this manuscript, using data from the study.

[1.] r= correlation coefficient, Δ%= difference between low and high fatty acid biomarker, β= beta-coefficient, PR = prevalence ratio

[2.] Adjustments: Santaren (2014)= demographics, lifestyle, dietary variables; Mozaffarian (2010)= age, sex, diabetes, smoking, coronary heart disease, physical activity, dietary intake (BMI, waist circumference); Mozaffarian (2013)= age, sex, race, education, smoking, alcohol intake, physical activity, prevalent diabetes, waist circumference; Nestel (2014)= age, systolic blood pressure,

waist:hip ratio; Oliveira (2013)= demographics, lifestyle, prevalent diabetes, medication; Kratz (2014)= age, sex, BMI; Castro-Webb (2012)= demographics, lifestyle, several fatty acids; Iggman (2010)= BMI, smoking, alcohol intake, physical activity; Smit (2010)= age, sex, residence, all trans-fatty acids, smoking, physical activity, income, diabetes, hypertension, energy intake and adipose tissue alpha-linolenic acid

[3.] + = An increase of the biomarker reduces the cardiovascular risk factor. - = An increase in the biomarker increases the cardiovascular risk factor. 0 = No significant effect

Abbreviations: AIRg, acute insulin response to glucose; AT, Adipose tissue; AUC, area under the curve for glucose; BMI, Body mass index; C14:0, Myristic acid; C15:0, Pentadecanoic acid; C17:0, Heptadecanoic acid; CE, Cholesterol esters; CRP, C-reactive protein; DBP, diastolic blood pressure; FFA, Free fatty acids; HC, Hip circumference; HDL, High density lipoprotein; HL, High level insulin infusion; HOMA-IR, homeostatic model assessment-estimated insulin resistance; IV DI, intravenous disposition index; LDL, Low density lipoprotein; LL, low-level insulin infusion; OGTT, oral glucose tolerance test; oral DI, oral disposition index; PL, Phospholipids; SBP, systolic blood pressure; SS, Subscapular Skinfold; SUS, Suprailiac skinfold; TG, Triglycerides; Trans-C16:1(n-7), Trans-Palmitoleic acid; TS, Triceps skinfold; WC, waist circumference.

Table S8B: Papers investigating the association of dairy fatty acid biomarkers with the development of cardiovascular diseases (based on an illustrative search)

								Biomarkers[1,2] (Risk (95%CI))			
**Paper**	**Population**	**n**	**Outcome**	**Blood Sample**	**Lipid Fraction**		**+/-[3]**	**C14:0**	**C15:0**	**C17:0**	**Trans-C16:1(n-7)**
			**Coronary Heart Disease**								
Iggman et al. (2016)[14]	Uppsala Longitudinal Study of Adult Men (ULSAM)	535 (251 cases)	Cardiovascular diseases	Adipose Tissue		HR[a]	0	0.95 (0.79 – 1.15)	1.03 (0.86-1.23)	0.98 (0.82-1.16)	
Khaw et al. (2012)[15]	Men and women from the EPIC cohort study	2424 cases, 4930 controls	Incidence of coronary heart disease	Plasma	PL	OR[a]	0	0.97 (0.87-1.08)	0.98 (0.89-1.09)	0.91 (0.82-1.01)	
Matthan et al. (2014)[16]	Post-menopausal women	2448 (1224 cases, 1224 controls)	Coronary heart disease	Plasma	PL	OR[a]	0	1.05 (0.70-1.56)	1.02 (0.61-1.72)		
Oliveira et al. (2013)[6]	US adults (45-84yr) from the Multi-ethnic study of athero-sclerosis	2837 (189 cases)	Incidence of cardiovascular disease	Plasma	PL	HR[b]	0	1.06 (0.65-1.74)	0.55 (0.32-0.95)		0.99 (0.58-1.69)
						HR[a]	+	1.02 (0.87-1.20)	0.81* (0.68-0.98)		0.97 (0.82-1.13)
		2837 (146 cases)	Incidence of coronary heart disease	Plasma	PL	HR[b]	+	1.13 (0.63-2.03)	0.41* (0.22-0.78)		0.82 (0.45-1.49)
						HR[a]	+	1.01 (0.84-1.21)	0.74* (0.60-0.92)		0.91 (0.75-1.10)
Sun et al. (2007)[17]	Female nurses, 30-55 years old	166 cases, 327 controls	Ischemic heart disease	Plasma	Total	RR[c]	-		2.36 * (1.16-4.78)	1.25 (0.68-2.30)	0.79 (0.44-1.42)
				Erythrocytes		RR[c]	0		0.93 (0.42-2.05)	0.72 (0.38-1.38)	0.98 (0.53-1.83)
Warensjö et al. (2010)[9]	Swedish general population	444 cases (307 men), 556 controls (308 men)	First myocardial infarction	Plasma	PL	OR[a] (men)	0		0.92 (0.78-1.10)	0.92 (0.78-1.10)	
				Plasma	PL	OR[a] (wo-men)	+		0.78* (0.61-0.99)	0.74* (0.58-0.95)	
			**Stroke**								
Warensjö et al. (2009)[18]	Northern Swedish general population	108 stroke cases, 216 controls	First stroke	Plasma	PL	OR[a]	+		0.81* (0.60-1.10)	0.71* (0.52-0.79)	
Yaemsiri et al. (2013)[19]	Womens Health Initiative	964 cases, 964 controls	Incident ischemic stroke	Serum	PL	OR[b,d]	0	1.09 (0.86-1.37)	1.07 (0.87-1.13)	1.01 (0.85-1.21)	0.94 (0.76-1.16)

	Observational Study (WHI-OS)										
Yakoob et al. (2014)[20]	Women in the Nurses' Health study (NHS) and men in the Health professionals study (HPFS)	472 cases NHS, 122 cases HPFS, 594 controls	Incident stroke	Plasma	Total	HR[e]	0	1.05 (0.62-1.78)	0.85 (0.54-1.33)	0.99 (0.67-1.49)	0.89 (0.55-1.45)
				Erythrocytes		HR[e]	0	1.31 (0.73-2.36)	0.88 (0.53-1.45)	0.87 (0.42-1.76)	1.07 (0.61-1.88)
Yamagishi et al. (2013)[21]	Men and Women from the Minneapolis Field Center of the Athero-sclerosis Risk in Communities Study (ARIC)	3870 (168 cases)	Incident Ischemic stroke	Plasma	CE	HR[e]	0	1.43 (0.92-2.22)	0.83 (0.55-1.25)		
					PL	HR[e]	0	1.29 (0.83-2.01)	0.89 (0.60-1.33)		
			**Heart Failure**								
Matsumoto et al. (2013)[22]	Physicians Health Study (PHS)	788 cases, 788 controls	Incident heart failure	Plasma	PL	OR[e]	0	1.03 (0.65-1.64)	0.73 (0.44-1.12)		
						OR[a]	0	1.02 (0.86-1.20)	0.91 (0.76-1.07)		
Tokede et al. (2013)[23]	Physicians Health Study (PHS)	788 cases, 788 controls	Incident heart failure	Plasma	PL	OR[b]	0				0.74 (0.49-1.10)
						OR[a]	0				0.91 (0.80-1.04)
Yamagishi et al. (2008)[24]	Men and Women from the Minneapolis Field Center of the Athero-sclerosis Risk in Communities Study (ARIC)	3592 (197 cases)	Incident heart failure	Plasma	CE	HR[e]	0	1.70** (1.06-2.71)	0.80 (0.50-1.28)	0.81 (0.52-1.26)	
				Plasma	PL	HR[e]	+	1.29 (0.80-2.08)	0.62* (0.38-1.02)	0.55** (0.35-0.85)	

Significance level: *= p <0.05, **= p <0.01, ***= p <0.001. One asterisk was ascribed when the risk was significant, but the level unknown.

[a]. Evaluated as continuous variables (per 1 SD unit difference)[b] . Highest versus lowest quintile [c.] Highest versus lowest tertile [d]. CI is 99.9% instead of 95%, [e] Highest versus lowest quartile.

[1.] RR= Relative risk, HR= hazard ratio, OR= Odds ratio
[2.] Adjustments: Iggman (2016)= age,sex and cardiovascular risk factors; Kwah (2012)= age, sex, polyunsaturated fatty acids, BMI, smoking, alcohol intake, physical activity, social class, education, blood pressure; Matthan (2014)= demographics, BMI, systolic blood pressure, smoking, education, medication and hormone use, family history of cardiovascular disease/stroke/myocardial infarction/type 2 diabetes, physical activity, dietary intake; de Oliveira (2013)= demographics, lifestyle, dietary variables; Sun (2007)= age, smoking, fasting status, BMI, postmenoposal status and hormone use, physical activity, alcohol intake, aspirin intake, history of hypertension, hypercholesterolemia, diabetes, linoleic acid, total trans fatty acids; Warensjo (2009) = BMI, serum-cholesterol, tobacco use, systolic blood pressure, diastolic blood pressure; Yaemsiri (2013)= BMI, smoking, diabetes, aspirin use, systolic blood pressure, antihypertensive medication, total cholesterol, HDL cholesterol and triglycerides; Yakoob (2014)= age, race, month of blood collection, smoking status, physical activity, alcohol, family history of diabetes, myocardial infarction, menoposal status, postmenoposal hormone use, food intake, energy intake, plasma/erythrocytes trans 18:1, trans 18:1, 16:0, 18:0; Yamagishi (2008 & 2013)= Age and sex; Matsumoto (2013) and Tokede (2013)= age, race, year of birth, blood sample collection, BMI, smoking, exercise, alcohol intake, history of diabetes and atrial fibrillation and saturated fatty acids.
[3.] + = An increase of the biomarker decreases the risk of diabetes type 2 and/or cardiovascular diseases. - = An increase in the biomarker increases the risk of diabetes type 2 and/or cardiovascular diseases. 0 = No significant effect

Abbreviations: AT, Adipose tissue; C14:0, Myristic acid; C15:0, Pentadecanoic acid; C17:0, Heptadecanoic acid; CE, Cholesterol esters; FFA, Free fatty acids; PL, Phospholipids; TG, Triglycerides; Trans-C16:1(n-7), Trans-Palmitoleic acid.

Table S8C: Papers investigating the association of dairy fatty acid biomarkers with the development of diabetes type 2 (based on an illustrative search)

							Biomarkers[1,2] (Risk (95%CI))			
**Paper**	**Population**	**n**	**Blood Sample**	**Lipid Fraction**		**+/-[3]**	**C14:0**	**C15:0**	**C17:0**	**Trans-C16:1(n-7)**
Forouhi et al. (2014)[25]	Adults in the EPIC cohort study	27296 (12132 cases, 15164 controls)	Plasma	PL	HR[a]	+	1.15* (1.09-1.22)	0.79* (0.73-0.85)	0.67* (0.63-0.71)	
Hodge et al. (2007)[26]	Adults in the Melbourne collaborative cohort study	364 cases, 3391 controls	Plasma	PL	OR[b]	+		0.26*** (0.17-0.40)		
Krachler et al. (2008)[27]	Adults in the VIP study	159 cases, 291 controls	Erythrocytes		OR[a]	+	1.25* (1.01-1.53)	0.65** (0.50-0.85)	0.47*** (0.35-0.65)	
Mozaffarian et al. (2010)[3]	Cardio-vascular Health study, adults	2985 (304 cases)	Plasma	PL	HR[b]	+				0.38*** (0.24-0.62)
					HR[c]	0		0.99 (0.89-0.10)	0.98 (0.85-1.13)	
Mozaffarian et al. (2013)[4]	US adults (45-84yr) from the Multi-ethnic study of athero-sclerosis	2281 (205 cases)	Plasma	PL	HR[b]	+	0.98 (0.63-1.53)	0.70 (0.42-1.15)		0.52* (0.32-0.85)
Santaren et al. (2014)[1]	Hispanic, African American and Non-Hispanic white adults 40-60 yr	659	Serum	Total	OR[a]	+		0.73* (0.56-0.95)		
Yakoob et al. (2016)[28]	Women in the Nurses' Health study (NHS) and men in the Health professionals study (HPFS)	3333 (184 cases NHS study, 93 cases HPFS study)	Plasma	Total	HR[d]	+	1.03 (0.65-1.64)	0.56* (0.37-0.86)	0.57* (0.39-0.83)	0.48*** (0.33-0.70)
		3289 (179 cases NHS study, 97 cases in HPFS study	Erythrocytes		HR[d]	+	1.56 (0.98-2.49)	0.83 (0.55-1.25)	0.54*** (0.40-0.73)	0.78* (0.51-1.18)

Significance level: *= p <0.05, **= p <0.01, ***= p <0.001. One asterisk was ascribed when the risk was significant, but the level unknown.

[a]. Evaluated as continuous variables (per 1 SD unit difference), [b.] Highest versus lowest quintile, [c]. HR's evaluated per 0.05% points of total fatty acids [d]. Highest versus lowest quartile

[1.] RR= Relative risk, HR= hazard ratio, OR= Odds ratio

[2.] Adjustments: Forouhi (2014), Santaren (2014),Mozaffarian (2010, 2013)= demographics, lifestyle, dietary variables; Hodge (2007)= demographic, diabetes history, physical activity, alcohol intake; Krachler (2008)= alcohol intake; Yakoob (2016)= age, race, month of blood collection, smoking status, physical activity, alcohol, family history of diabetes, myocardial infarction, menoposal status, postmenoposal hormone use, food intake, energy intake, plasma/erythrocytes trans 18:1, trans 18:1, 16:0, 18:0;
[3.] + = An increase of the biomarker decreases the risk of diabetes type 2 and/or cardiovascular diseases. - = An increase in the biomarker increases the risk of diabetes type 2 and/or cardiovascular diseases. 0 = No significant effect

Abbreviations: AT, Adipose tissue; C14:0, Myristic acid; C15:0, Pentadecanoic acid; C17:0, Heptadecanoic acid; CE, Cholesterol esters; FFA, Free fatty acids; PL, Phospholipids; TG, Triglycerides; Trans-C16:1(n-7), Trans-Palmitoleic acid.

References

1. Santaren ID, Watkins SM, Liese AD, et al. Serum pentadecanoic acid (15:0), a short-term marker of dairy food intake, is inversely associated with incident type 2 diabetes and its underlying disorders. *Am J Clin Nutr*. 2014;100(6):1532-1540.

2. Smedman AE, Gustafsson IB, Berglund LG, Vessby BO. Pentadecanoic acid in serum as a marker for intake of milk fat: Relations between intake of milk fat and metabolic risk factors. *Am J Clin Nutr*. 1999;69(1):22-29.

3. Mozaffarian D, Cao H, King IB, et al. Trans-palmitoleic acid, metabolic risk factors, and new-onset diabetes in U.S. adults: A cohort study. *Ann Intern Med*. 2010;153(12):790-799.

4. Mozaffarian D, de Oliveira Otto MC, Lemaitre RN, et al. Trans-palmitoleic acid, other dairy fat biomarkers, and incident diabetes: The multi-ethnic study of atherosclerosis (MESA). *Am J Clin Nutr*. 2013;97(4):854-861.

5. Nestel PJ, Straznicky N, Mellett NA, et al. Specific plasma lipid classes and phospholipid fatty acids indicative of dairy food consumption associate with insulin sensitivity. *Am J Clin Nutr*. 2014;99(1):46-53.

6. de Oliveira Otto MC, Nettleton JA, Lemaitre RN, et al. Biomarkers of dairy fatty acids and risk of cardiovascular disease in the multi-ethnic study of atherosclerosis. *J Am Heart Assoc*. 2013;2(4):e000092.

7. Kratz M, Marcovina S, Nelson JE, et al. Dairy fat intake is associated with glucose tolerance, hepatic and systemic insulin sensitivity, and liver fat but not beta-cell function in humans. *Am J Clin Nutr*. 2014;99(6):1385-1396.

8. Rosell M, Johansson G, Berglund L, Vessby B, de Faire U, Hellenius ML. The relation between alcohol intake and physical activity and the fatty acids 14 : 0, 15 : 0 and 17 : 0 in serum phospholipids and adipose tissue used as markers for dairy fat intake. *Br J Nutr*. 2005;93(1):115-121.

9. Warensjo E, Jansson JH, Cederholm T, et al. Biomarkers of milk fat and the risk of myocardial infarction in men and women: A prospective, matched case-control study. *Am J Clin Nutr*. 2010;92(1):194-202.

10. Castro-Webb N, Ruiz-Narvaez EA, Campos H. Cross-sectional study of conjugated linoleic acid in adipose tissue and risk of diabetes. *Am J Clin Nutr*. 2012;96(1):175-181.

11. Iggman D, Arnlov J, Vessby B, Cederholm T, Sjogren P, Riserus U. Adipose tissue fatty acids and insulin sensitivity in elderly men. *Diabetologia*. 2010;53(5):850-857.

12. Rosell M, Johansson G, Berglund L, Vessby B, de Faire U, Hellenius ML. Associations between the intake of dairy fat and calcium and abdominal obesity. *Int J Obes Relat Metab Disord*. 2004;28(11):1427-1434.

13. Smit LA, Willett WC, Campos H. Trans-fatty acid isomers in adipose tissue have divergent associations with adiposity in humans. *Lipids*. 2010;45(8):693-700.

14. Iggman D, Arnlov J, Cederholm T, Riserus U. Association of adipose tissue fatty acids with cardiovascular and all-cause mortality in elderly men. *JAMA Cardiol*. 2016;1(7):745-753.

15. Khaw KT, Friesen MD, Riboli E, Luben R, Wareham N. Plasma phospholipid fatty acid concentration and incident coronary heart disease in men and women: The EPIC-norfolk prospective study. *PLoS Med*. 2012;9(7):e1001255.

16. Matthan NR, Ooi EM, Van Horn L, Neuhouser ML, Woodman R, Lichtenstein AH. Plasma phospholipid fatty acid biomarkers of dietary fat quality and endogenous metabolism predict coronary heart disease risk: A nested case-control study within the women's health initiative observational study. *J Am Heart Assoc*. 2014;3(4):10.1161/JAHA.113.000764.

17. Sun Q, Ma J, Campos H, Hu FB. Plasma and erythrocyte biomarkers of dairy fat intake and risk of ischemic heart disease. *Am J Clin Nutr*. 2007;86(4):929-937.

18. Warensjo E, Smedman A, Stegmayr B, et al. Stroke and plasma markers of milk fat intake--a prospective nested case-control study. *Nutr J*. 2009;8:21-2891-8-21.

19. Yaemsiri S, Sen S, Tinker LF, et al. Serum fatty acids and incidence of ischemic stroke among postmenopausal women. *Stroke*. 2013;44(10):2710-2717.

20. Yakoob MY, Shi P, Hu FB, et al. Circulating biomarkers of dairy fat and risk of incident stroke in U.S. men and women in 2 large prospective cohorts. *Am J Clin Nutr*. 2014;100(6):1437-1447.

21. Yamagishi K, Folsom AR, Steffen LM, ARIC Study Investigators. Plasma fatty acid composition and incident ischemic stroke in middle-aged adults: The atherosclerosis risk in communities (ARIC) study. *Cerebrovasc Dis*. 2013;36(1):38-46.

22. Matsumoto C, Hanson NQ, Tsai MY, Glynn RJ, Gaziano JM, Djousse L. Plasma phospholipid saturated fatty acids and heart failure risk in the physicians' health study. *Clin Nutr*. 2013;32(5):819-823.

23. Tokede OA, Petrone AB, Hanson NQ, et al. Plasma phospholipid trans fatty acids and risk of heart failure. *Am J Clin Nutr*. 2013;97(4):698-705.

24. Yamagishi K, Nettleton JA, Folsom AR, ARIC Study Investigators. Plasma fatty acid composition and incident heart failure in middle-aged adults: The atherosclerosis risk in communities (ARIC) study. *Am Heart J*. 2008;156(5):965-974.

25. Forouhi NG, Koulman A, Sharp SJ, et al. Differences in the prospective association between individual plasma phospholipid saturated fatty acids and incident type 2 diabetes: The EPIC-InterAct case-cohort study. *Lancet Diabetes Endocrinol*. 2014;2(10):810-818.

26. Hodge AM, English DR, O'Dea K, et al. Plasma phospholipid and dietary fatty acids as predictors of type 2 diabetes: Interpreting the role of linoleic acid. *Am J Clin Nutr*. 2007;86(1):189-197.

27. Krachler B, Norberg M, Eriksson JW, et al. Fatty acid profile of the erythrocyte membrane preceding development of type 2 diabetes mellitus. *Nutr Metab Cardiovasc Dis*. 2008;18(7):503-510.

28. Yakoob MY, Shi P, Willett WC, et al. Circulating biomarkers of dairy fat and risk of incident diabetes mellitus among men and women in the united states in two large prospective cohorts. *Circulation*. 2016;133(17):1645-1654.
